# The Essential Oil Composition and Antimicrobial Activity of *Liquidambar formosana* Oleoresin

**DOI:** 10.3390/plants9070822

**Published:** 2020-06-30

**Authors:** Anjanette DeCarlo, Tao Zeng, Noura S. Dosoky, Prabodh Satyal, William N. Setzer

**Affiliations:** 1 Aromatic Plant Research Center, 230 N 1200 E Suite 100, Lehi, UT 84043, USA; ndosoky@aromaticplant.org (N.S.D.); psatyal@aromaticplant.org (P.S.); 2 College of Chemical Engineering, Nanjing Forestry University, Nanjing 210037, China; zengtao@njfu.edu.cn; 3 Department of Chemistry, University of Alabama in Huntsville, Huntsville, AL 35899, USA

**Keywords:** sweetgum, chemical composition, (*E*)-caryophyllene, α-pinene, β-pinene, camphene, enantiomeric distribution, hierarchical cluster analysis, principal component analysis, antibacterial, antifungal

## Abstract

The oleoresin essential oils of *Liquidambar formosana* have potential therapeutic benefits. However, current research on *L. formosana* oleoresin essential oil is still in its early stages, and its chemotypic characterization is undefined. For better leveraging of plant resources and application of the essential oil, we collected 25 *L. formosana* oleoresin essential oil samples of individual trees from different geographical areas of Southern China. The essential oils were obtained by hydrodistillation and analyzed by gas chromatography–mass spectrometry (GC–MS) and gas chromatography–flame ionization detection (GC–FID). The major components of the essential oils were (*E*)-caryophyllene (3.3%-64.4%), α-pinene (0.6%-34.5%), β-pinene (0.6%-26.0%), camphene (0.3%-17.3%), and limonene (0.2%-7.9%). A chiral GC–MS analysis was carried out on the essential oil samples and (–)-α-Pinene, (–)-β-pinene, (–)-camphene, and (–)-limonene were the dominant enantiomers in *L. formosana* essential oil. The chemical categories of *L. formosana* oleoresin essential oils were clarified by agglomerative hierarchical cluster analysis (AHC) and principal component analysis (PCA). The multivariate analyses demonstrated that a total of four chemical groups can be delineated for *L. formosana*. The *L. formosana* essential oils were screened for antimicrobial activity against a panel of potentially pathogenic bacteria and fungi and showed promising antimicrobial activities with minimum inhibitory concentration (MIC) ≤ 625 μg/mL. These results highlight the economic value of *L. formosana* oleoresin essential oil, the importance of *L. formosana* sustainability, and the potential therapeutic benefits of its oleoresin essential oils.

## 1. Introduction

In recent years, essential oils or resins from aromatic plants have been widely applied in the food, cosmetic, and medicinal industries. Research and the related applications of aromatic plants play a more and more important role in preserving biodiversity, encouraging agroecology, and helping social and environmental development. The genus *Liquidambar* L. is one category of aromatic plants; it includes five species, in which two species and one variety are found in China. *Liquidambar formosana* Hance is one of the species in the Hamamelidaceae family [1,2]. Known for its bright orange autumn leaves, *L. formosana* is a large, flowering, deciduous tree.

The fruit of *L. formosana* (Chinese name LuLuTong) has been used as a traditional Chinese medicine for thousands of years [3]; the pharmacology and phytochemistry of *L. formosana* has been reviewed [4]. The fruit extract of *L. formosana* has shown anti-inflammatory activity [5]. The fruits of *L. formosana* have yielded lupane and oleanane triterpenoids [6]. The fruit essential oil has shown antifungal activity and was dominated by α-pinene (16.8%), (*E*)-caryophyllene (10.1%), τ-muurolol (8.3%), τ-cadinol (7.6%), β-pinene (6.7%), and sabinene (5.7%) [7].

The leaves of *L. formosana* are a source of lignan glycosides and flavonoid glycosides as well as hydrolysable tannins [6,8], which is consistent with the antioxidant activity of *L. formosana* leaf extracts [9,10]. The leaf essential oil of *L. formosana* from Taiwan has shown α-pinene (40.9%), β-pinene (24.8%), limonene (18.3%), α-phellandrene (8.9%), terpinen-4-ol (8.1%), and sabinene (5.6%) as the major components [11]. In contrast, another leaf essential oil sample from Taiwan had terpinen-4-ol (32.0%), β-pinene (18.0%), γ-terpinene (13.8%), and α-terpinene (9.7%) as the predominant compounds [12].

The trunk of *L. formosana* can secrete an oleoresin due to insects, artificial tapping, or other mechanical damage. *L. formosana* oleoresin is made up of a solid resin component and a volatile component. The essential oil from the oleoresin can be obtained by hydrodistillation. *L. formosana* resin has numerous medical applications in Asian folk medicine, such as being a promoter of blood circulation, an alleviator of blood stasis, an analgesic, and an anti-inflammatory and wound-healing agent [3,4]. *L. formosana* oleoresin has been the source of several abietane diterpenoids [13], oleanane and lupane triterpenoids with cytotoxic [14], antifungal [15], glycogen phosphorylase inhibitory [16], and nuclear factor of activated T cell (NFAT) transcription factor inhibitory [17] activities. Chen et al. have reported the toxicity and antibacterial efficacy of *L. formosana* [18]. The main components of essential oil are the monoterpenes α-pinene (22.4–34.1%), β-pinene (15.7–23.8%), myrcene (up to 10.9%), and limonene (3.4–8.4%); and the sesquiterpene (*E*)-caryophyllene (7.8–26.5%) [15,19]. These components suggest that *L. formosana* oleoresin essential oil also has potential therapeutic benefits for human health.

*Liquidambar formosana* is widely distributed in China, mainly in the Yellow River Basin and further south. However, there is a lack of fundamental research on the development of *L. formosana* as a source of non-timber forest products (NTFP), which has limited the comprehensive utilization of this species. This has resulted in little production of *L. formosana* oleoresin and low oleoresin product quality. Currently, many *L. formosana* trees have been harvested for timber or for pulp [20], or they are cleared to allow for grazing [21], which is detrimental to the environment. Although they are an important part of the primary forest, according to our field observations, some old *L. formosana* trees have been cut down and their growing areas converted into cultivated land. As a renewable NTFP, the oleoresin collection from *L. formosana* represents a more eco-friendly practice by preserving old-growth forests while still providing economic incentives for local people. For the sustainable development of *L. formosana* plant resources, efforts have been made to develop methods that not only protect the ecology but also increase the oleoresin yield [22]. The few comprehensive analyses of the potential chemotypes of *L. formosana* oleoresin essential oils have not revealed any information about the quality of the oleoresin oils or explained whether the oils had been adulterated. In this work, we present the chemical characterization of *L. formosana* oleoresin essential oils, multivariate analyses to define potential chemotypes, and the screening of the oleoresin essential oils for activity against bacteria and fungi of dermatological or pulmonary importance.

## 2. Results and Discussion

### 2.1. Essential Oil Chemical Composition

*Liquidambar formosana* oleoresins were collected from 25 individual trees from several locations in Southern China (i.e., Leye, Longsheng, and Wangmo). These areas are geologically the same; they consist of limestone mountains with karst formations. In general, secondary growth, dominated by younger even-aged trees with low biodiversity, were found in the Wangmo area, whereas the Leye area had old growth trees in naturally biodiverse remnant forest patches. The amount of resin collected from each tree is given in Table 1. The oleoresin samples were hydrodistilled to give essential oils in yields ranging from 1.54% to 30.2% (Table 1). Each of the essential oils was analyzed by gas chromatography–mass spectrometry (GC–MS) and gas chromatography–flame ionization detection (GC–FID) (three replicates per essential oil sample) (Table 2). A total of 191 compounds were identified in the essential oils, accounting for 98.1–99.9% of the compositions. The major components in the essential oils were (*E*)-caryophyllene (3.3–64.4%), α-pinene (0.6–34.5%), β-pinene (0.6–26.0%), camphene (0.3–17.3%), and limonene (0.2–7.9%).

The results from the present study (Supplementary Table S1) are in qualitative agreement with previous studies on the oleoresin essential oil. Liu and Chen reported α-pinene (24.9%), β-pinene (23.6%), (*E*)-caryophyllene (19.6%), and camphene (8.8%) as the major components in the oleoresin essential oil [23]. Similarly, Chien and co-workers carried out a solid-phase microextraction (SPME) and found the oleoresin volatiles to have α-pinene (23.3%), (*E*)-caryophyllene (22.7%), β-pinene (19.6%), myrcene (10.9%), and limonene (8.4%) [15]. Song and Zeng obtained oleoresin essential oils from three different sites (Jiangxi, Guangxi, and Guizhou provinces) [19]. These workers found the oleoresin essential oils to be composed largely of α-pinene (34.1%, 33.5%, 22.4%), β-pinene (21.4%, 23.8%, 15.7%), camphene (14.1%, 7.7%, 7.3%), limonene (7.9%, 4.1%, 3.4%), and (*E*)-caryophyllene (7.8%, 8.9%, 26.5%). The present work, however, is much expanded compared to previous examinations of *L. formosana* oleoresin essential oil. In this work, the diameter at breast height (dbh), global positioning system (gps) coordinates, and elevation for 25 individual trees were measured and the oleoresins obtained from each tree. The essential oils were obtained and a more thorough chemical analysis carried out, revealing a total of 191 compounds identified in the essential oils, in order to assess any correlation between these data and the essential oils’ chemical profiles.

**Figure 1:**
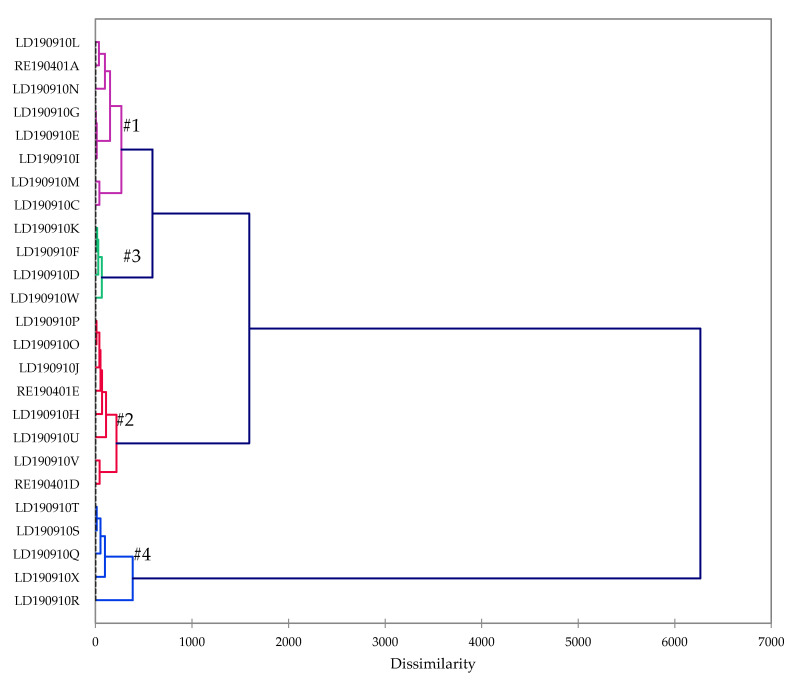
Dendrogram obtained from the agglomerative hierarchical cluster analysis of 25 *Liquidambar formosana* oleoresin essential oil compositions.

**Figure 2:**
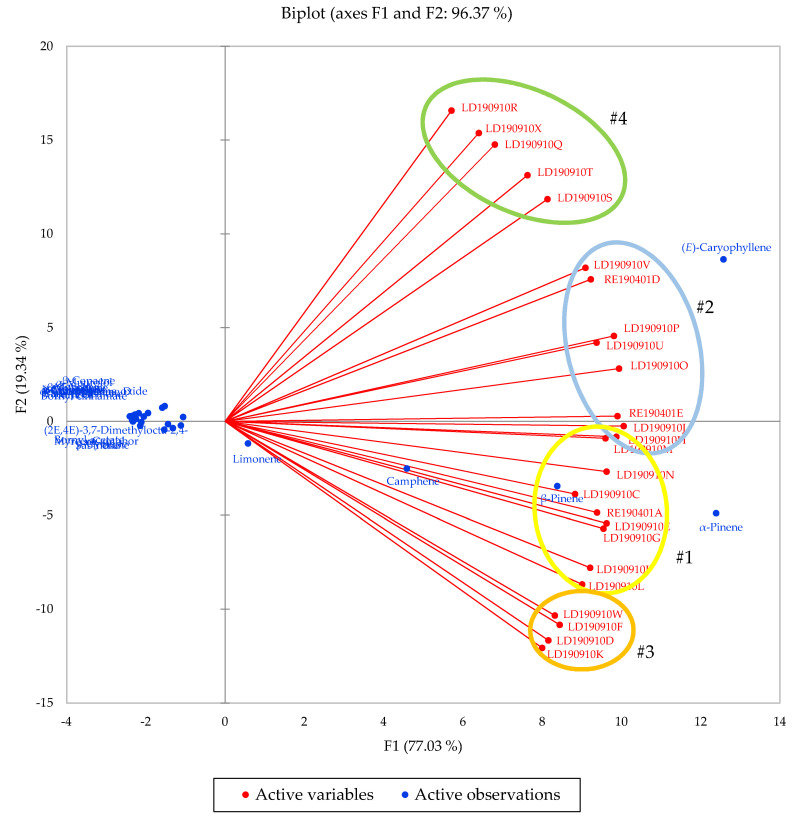
Principal component biplot of PC1 and PC2 scores and loadings indicating the chemical groupings of *Liquidambar formosana* based on compositions of the oleoresin essential oils.

Agglomerative hierarchical cluster (AHC) analysis revealed four clearly defined groups (Figure 1, Table 2). Group #1 was dominated by α-pinene (18.8–27.8%), β-pinene (12.0–20.8%), (*E*)-caryophyllene (8.9–18.5%), and camphene (9.2–15.3%). Group #2 had the same major components in a different order of concentration: (*E*)-caryophyllene (19.9–34.8%), α-pinene (15.3–25.4%), β-pinene (10.9–18.7%), and camphene (6.0–10.9%). Group #3 was rich in α-pinene (29.1–34.5%), β-pinene (20.6–26.0%), and camphene (11.0–17.3%), while group #4 was dominated by (*E*)-caryophyllene (42.0–64.4%). A biplot from the principal component analysis (Figure 2) shows the associations between the clusters and the major components.

There is no obvious correlation between the oleoresin essential oil chemistry from the cluster analysis and the geographical location of the collections, but there are some trends. Most of the trees from the Wangmo collections were dominated by (*E*)-caryophyllene (groups #2 and #4) and most of the trees from the Leye collection sites were dominated by pinenes (groups #1 and #3). Interestingly, however, adjacent trees (Leye 8 and Leye 9) fell into different clusters (#1 and #2, respectively). Likewise, adjacent trees Leye 6 and Leye 7 were also in different groups (#1 and #3), although both of these groups were dominated by pinenes. Three trees, Wangmo 23, 24, and 25, were collected from the same general area and all showed different chemistries; Wangmo 23 fell into group #2, Wangmo 24 into group #3, and Wangmo 25 into group #4. Furthermore, there does not seem to be a correlation between the time of year (March vs. August) in the observed oleoresin essential oil composition. Thus, for example, samples RE190401D and RE190401E, both collected from Wangmo in March 2019, fell into group #2, along with four Wangmo samples collected in August 2019 (LD190910O, LD190910P, LD190910U, and LD190910V). Similarly, there is little correlation between tree size and oleoresin essential oil composition. Oleoresin essential oils from the largest trees (i.e., LD190910D, LD190910K, and LD190910N) were either in group #1 or group #3 (pinene-rich groups), but one of the smallest trees (LD190910W) also yielded a pinene-rich essential oil (group #3). This suggests that there are no major differences in regard to collection site, size of tree, or collection time of year.

A chiral GC–MS analysis was carried out on the *L. formosana* oleoresin essential oils (Supplementary Table S2). For both α-pinene and β-pinene, the (–)-enantiomer predominated (65.1–95.6% and 56.7–97.6%, respectively). Limonene was exclusively (–)-limonene while camphor was exclusively (+)-camphor. The (+)-enantiomers also dominated α-thujene and sabinene, while the (–)-enantiomers dominated camphene, borneol, and bornyl acetate. There are no obvious correlations between the enantiomeric distributions of monoterpenoids and the geographical location, size of tree, or time of year of the collection.

### 2.2. Antibacterial and Antifungal Activity

The *L. formosana* essential oils were screened for antimicrobial activity against a panel of potential dermal and pulmonary pathogenic bacteria (*Bacillus cereus*, *Cutibacterium acnes*, *Staphylococcus aureus*, *Staphylococcus epidermidis*, *Streptococcus pyogenes*, *Pseudomonas aeruginosa*, and *Serratia marcescens*) (Table 3) and fungi (*Aspergillus fumigatus*, *Aspergillus niger*, *Microsporum canis*, *Microsporum gypseum*, *Trichophyton mentagrophytes*, *Trichophyton rubrum*, and *Candida albicans*) (Table 4). All of the tested essential oil samples demonstrated similar antibacterial and antifungal profiles, which is not surprising considering the similarity of the essential oil compositions. Sartoratto and co-workers have suggested that essential oils showing minimum inhibitory concentration (MIC) values < 500 μg/mL are strong inhibitors, while those with MIC values of 600–1500 μg/mL are moderate inhibitors [24]. Based upon these criteria, the *L. formosana* oleoresin essential oils showed strong antimicrobial activity against all organisms except for *S. marcescens* (MIC = 625 μg/mL). In particular, the oil of *L. formosana* showed excellent antibacterial activity against *S. epidermidis* (MIC = 78 μg/mL) and strong antifungal activity against *A. niger* (MIC = 78–313 μg/mL). The mechanisms for antimicrobial activities of essential oils are not completely understood. However, it has been suggested that the lipophilic essential oil components serve to disrupt and penetrate the lipid structure of the cell wall, increasing membrane fluidity and causing the leakage of H_3_O^+^ and K^+^ ions, ultimately leading to cell lysis and death [25,26].

In general, the oleoresin essential oils showed better antibacterial activity against Gram-positive organisms over Gram-negative organisms. Except for *S. epidermidis* (MIC = 78 μg/mL), the antibacterial activity of *L. formosana* oleoresin essential oils against Gram-positive organisms was 156 μg/mL, whereas the MIC values against Gram-negative *P. aeruginosa* were 313 μg/mL and 625 μg/mL against *S. marcescens*. It has often been observed that Gram-negative bacteria are less susceptible to the inhibitory effects of essential oils than Gram-positive bacteria [27,28,29]. This phenomenon has been attributed to the presence of cell wall lipopolysaccharides in the Gram-negative organisms, which can inhibit the lipophilic essential oil components from diffusing into the cells [30].

The antibacterial activity observed for the *L. formosana* oleoresin essential oils against *S. epidermidis* cannot be attributed solely to the activities of the individual major components (Table 3). Thus, for example, (*E*)-caryophyllene, α-pinene, β-pinene, camphene, and (+)-limonene have MIC values of 313 μg/mL against *S. epidermidis*, and only (–)-limonene has an MIC value of 78 μg/mL. Similarly, the MIC value for *L. formosana* oleoresin essential oils against *S. pyogenes* was 156 μg/mL, but the MIC values for the major components ranged from 313 μg/mL to 625 μg/mL. It is likely that the synergistic effects of the major components, possibly involving minor components, are responsible for the antimicrobial activity [31,32]. Crevelin and co-workers observed synergistic antimicrobial effects in a combination of α-pinene, β-pinene, (*E*)-caryophyllene, and caryophyllene oxide [33]. Both α-pinene and β-pinene showed good antifungal activity against *A. niger*, and these components may be responsible for the activity of *L. formosana* essential oil against this fungal organism. (*E*)-Caryophyllene, on the other hand, was inactive against *A. niger*.

## 3. Materials and Methods

### 3.1. Oleoresin Collection

**Figure 3:**
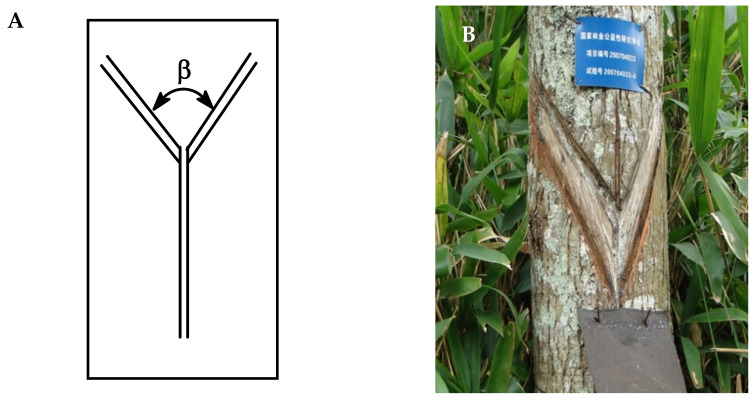
Illustration of the V-shaped downward tapping method. **A**: Schematic diagram, **B**: Photograph of appropriately tapped *L. formosana*.

*Liquidambar formosana* oleoresins were collected from 25 individual trees from several locations in Southern China (Table 1). The oleoresin tapping practice that was utilized in this work not only increases resin tapping efficiency but also ensures the sustainability of the trees and the ecosystem. It was invented by Zeng and co-workers and named the “downward tapping method of V-shaped” [22]. More specifically, mature *L. formosana* trees with DBH (diameter at breast height) ≥ 60 cm, without pest infection, qualified for oleoresin tapping. The oleoresin tapping was carried out by local farmers at average temperatures above 15 °C. The tapping surface was on the sun-facing side of the trunk. An area of the trunk was shaved of bark, a medial groove was cut in the center of the shaved area, and a V-shaped ditch was cut from top to bottom along the vertical direction of the trunk. The V-shaped angle (β) was between 60° and 80° (Figure 3) and was cut to the first or second annual ring of the tree. A minimum 10-cm space was preserved between each V-shaped ditch to ensure the health of the trees. The oleoresin aggregations were located at the bottom of the V-shaped ditch, which guided the oleoresin into a container. Immediately after the resin was collected, it was transferred to amber-colored glass bottles and stored at 4 °C until distillation.

### 3.2. Oleoresin Hydrodistillation

The hydrodistillation of the samples of *L. formosana* oleoresin was performed with an all-glass Clevenger apparatus for 7 h. The water and resin were mixed in a ratio of 6:1 and the hydrodistillation was carried out with constant stirring of the mixture. The rate of hydrodistillation was around 2 mL/min. The isolated oil had a strong resinous aroma with floral, pine, and spicy notes. Oleoresin masses and essential oil yields are summarized in Table 1. The *L. formosana* oleoresin essential oils were stored in sealed amber vials at 4 °C until chromatographic analysis and bioactivity screening.

### 3.3. Gas Chromatographic–Mass Spectral Analysis

The oleoresin essential oils from *L. formosana* were subjected to gas chromatographic–mass spectral (GC–MS) analysis, as previously reported [34]: Shimadzu GCMS-QP2010 Ultra, electron impact (EI) mode with electron energy = 70 eV, scan range = 40–400 atomic mass units, scan rate = 3.0 scans/s, and Shimadzu GC-MS solution software v. 4.45 (Shimadzu Scientific Instruments, Columbia, MD, USA); ZB-5ms fused silica capillary GC column Phenomenex, Torrance, CA, USA; (5% phenyl)-polymethylsiloxane stationary phase, 0.25 μm film thickness; helium carrier gas, column head pressure = 552 kPa, flow rate = 1.37 mL/min; injector temperature = 260 °C, ion source temperature = 260 °C; GC oven temperature program: initial temperature = 50 °C, temperature increased 2 °C/min to 260 °C. For each sample, a 5% *w*/*v* solution in CH_2_Cl_2_ was prepared, and 0.1 μL was injected using a split ratio of 30:1. Identification of the individual components of the essential oils was determined by comparison of the Kovats retention indices, determined using a series of *n*-alkanes, in addition to comparison of the mass spectral fragmentation patterns with those found in the MS databases [35,36,37,38], using the LabSolutions GCMS solution software version 4.45 (Shimadzu Scientific Instruments, Columbia, MD, USA) and with matching factors >90%.

### 3.4. Gas Chromatographic–Flame Ionization Detection

Quantitative analysis of the *L. formosana* essential oils was carried out by GC–FID, as previously reported [34]: Shimadzu GC 2010 equipped with FID, a split/splitless injector, and Shimadzu autosampler AOC-20i (Shimadzu Scientific Instruments, Columbia, MD, USA), with a ZB-5 capillary column (Phenomenex, Torrance, CA, USA). The GC–FID measurements were carried out using the same oven temperature program as that for GC–MS. Injector temperature = 250 °C, detector temperature = 280 °C, and nitrogen was the carrier gas, with a flow rate of 1.0 mL/min. The concentrations of the oleoresin essential oil components were calculated from raw peak areas, normalized to 100%, without standardization.

### 3.5. Chiral Gas Chromatography–Mass Spectrometry

Chiral GC–MS of the *L. formosana* oleoresin essential oils was carried out, as reported previously [34]: Shimadzu GCMS-QP2010S (Shimadzu Scientific Instruments, Columbia, MD, USA), electron impact (EI) mode, electron energy = 70 eV; scan range = 40–400 amu, scan rate = 3.0 scans/s; Restek B-Dex 325 chiral capillary GC column (Restek Corp., Bellefonte, PA, USA) (30 m × 0.25 mm ID × 0.25 μm film thickness). Oven temperature program: starting temperature = 50 °C, temperature increased 1.5 °C/min to 120 °C, then 2 °C/min to 200 °C, and kept at 200 °C for an additional 5 min; carrier gas was helium, flow rate = 1.8 mL/min. For each essential oil sample, a 3% *w*/*v* solution in CH_2_Cl_2_ was prepared, and 0.1 μL was injected using a split ratio of 1:45. The enantiomers of the monoterpenoids were identified by comparison of retention times with authentic samples obtained from Sigma-Aldrich (Milwaukee, WI, USA). The enantiomer percentages were determined from peak areas.

### 3.6. Antimicrobial Screening

The essential oils were screened for antibacterial activity against Gram-positive bacteria (*Bacillus cereus* (ATCC No. 14579), *Cutibacterium acnes* (ATCC No. 11827), *Staphylococcus aureus* (ATCC No. 29213), *Staphylococcus epidermidis* (ATCC No. 12228), and *Streptococcus pyogenes* (ATCC No. 19615)) and Gram-negative bacteria (*Pseudomonas aeruginosa* (ATCC No. 27853) and *Serratia marcescens* (ATCC No. 14756)), and for antifungal activity against molds (*Aspergillus niger* (ATCC No. 16888), *Aspergillus fumigatus* (ATCC No. 96918), *Microsporum canis* (ATCC No. 11621), *Microsporum gypseum* (ATCC No. 24102), and *Trichophyton mentagrophytes* (ATCC No. 18748)), and one yeast (*Candida albicans* (ATCC No. 18804)) using the microbroth dilution technique [39,40], as previously reported [41,42]. Individual essential oil components, ((*E*)-caryophyllene, (+)-α-pinene, (–)-α-pinene, (–)-β-pinene, camphene, (+)-limonene, and (–)-limonene, were obtained from Sigma-Aldrich (St. Louis, MO) and were used as received, without additional purification.

All bacteria were cultured on tryptic soy agar, except for *C. acnes*, which was grown on tryptic soy agar supplemented with 7% (*v*/*v*) defibrinated whole sheep blood (Cleveland Scientific, Bath, Ohio, USA), under micro-aerophilic conditions [43]. All fungi were cultured on yeast malt agar (Sigma-Aldrich, St. Louis, MO). For the bacteria and fungi, 50 μL of 1% (w/v) solution of the samples in dimethyl sulfoxide (DMSO) was diluted in 50 μL of cation-adjusted Mueller Hinton broth (CAMHB) (Sigma-Aldrich, St. Louis, MO). The sample solutions were then serially diluted (1:1) in fresh CAMHB to obtain concentrations of 2500, 1250, 625, 313, 156, 78, 39, and 20 μg/mL. The microbes were harvested from a fresh culture and added to each well at a concentration of approximately 1.5 × 10^8^ CFU/mL for bacteria and 7.5 × 10^7^ CFU/mL for fungi, and the 96-well microdilution plates for bacteria were incubated at 37 °C and the fungi were incubated at 35 °C for 24 h. The minimum inhibitory concentration (MIC) was determined as the lowest concentration with no turbidity. Gentamicin (Sigma-Aldrich, St. Louis, MO) was used as a positive antibiotic control and DMSO was used as the negative control (50 μL DMSO diluted in 50 μL broth medium and then serially diluted, as above). For fungi, the above-mentioned method was implemented using a yeast-nitrogen base growth medium (Sigma-Aldrich, St. Louis, MO, USA) and amphotericin B (Sigma-Aldrich, St. Louis, MO, USA) as a positive antifungal control.

### 3.7. Statistical Analysis

Multivariate analyses were carried out on the essential oil compositions of the *L. formosana* oleoresins. The chemical compositions of the 25 essential oils were treated as operational taxonomic units (OTUs), and the percentage compositions of 25 major components were used to ascertain the associations between the oleoresin essential oil compositions using agglomerative hierarchical cluster (AHC) analysis, using XLSTAT Premium, version 2018.5.53172 (Addinsoft, Paris, France). The dissimilarity of the samples was evaluated using Euclidean distance, and the clusters were defined using Ward’s method [44]. The principal component analysis (PCA) was carried out using the 25 major chemical components as variables, with a Pearson correlation matrix, using XLSTAT Premium, version 2018.1.1.60987 (Addinsoft, Paris, France). In all cases, 625 data (25 samples × 25 variables) were utilized for the principal component analysis.

## 4. Conclusions

*L. formosana* trees and their essential oils are important non-timber forest product (NTFP) resources. Due to the lack of research on *L. formosana*, the resources are under-utilized or even being destroyed. The oleoresin essential oils collected from 25 different *L. formosana* trees from different regions of Southern China showed very little variation in either chemical composition or enantiomeric distribution. The essential oil yields ranged from 7.7% to 30.2%. The oleoresin essential oils showed promising antibacterial efficacy against Gram-positive bacteria and antifungal activity. The biological potency, coupled with improved tree-tapping methods, promoted *L. formosana* oleoresin essential oil in terms of its economic potential as well as its therapeutic benefits. This is an innovative research work that extends our understanding of the phytochemistry of this tree as well as providing scientific and practical support for the development and utilization of *L. formosana* tree resources. As a new NTFP product, the oleoresin essential oils also improve awareness of ecosystem protection.

## Figures and Tables

**Table 1:** *Liquidambar formosana* oleoresin collection details.

Sample Code	Collection Site	dbh, cm	GPS Coordinates	Collection Date	Oleoresin Mass (g)	Essential Oil Yield (%)
RE190401A	Longsheng (tree A)	170	25°41′51.70”N, 110° 9′8.80”E, elev 1204 m	3/16/2019	23.10	11.43
RE190401D	Wangmo (tree C)	105	25°22′35.22”N, 106° 2′6.54”E, elev 573 m	3/17/2019	173.60	13.61
RE190401E	Wangmo (tree F)	87	25°24′32.88”N, 105°58′23.16”E, elev 780 m	3/17/2019	92.00	18.00
LD190910C	4 Leye	200	24°37′36.23”N, 106°33′25.27”E, elev 921 m	8/27/2019	55.10	23.96
LD190910D	5 Leye	303	24°37′36.23”N, 106°33′25.26”E, elev 924 m	8/27/2019	41.20	14.20
LD190910E	6 Leye	150	24°52′56.10”N, 106°26′20.36”E, elev 1111 m	8/27/2019	49.40	19.98
LD190910F	7 Leye	190	24°52′56.10”N, 106°26′20.36”E, elev 1111 m	8/27/2019	40.54	30.17
LD190910G	8 Leye	158	24°52′58.85”N, 106°25′52.28”E, elev 1213 m	8/27/2019	52.80	17.92
LD190910H	9 Leye	144	24°52′58.85”N, 106°25′52.28”E, elev 1213 m	8/27/2019	40.00	17.53
LD190910I	10 Leye	187	24°52′59.58”N, 106°26′19.95”E, elev 1127 m	8/27/2019	38.80	23.30
LD190910J	11 Leye	94	24°47′20.39”N, 106°34′28.89”E, elev 1118 m	8/27/2019	55.34	17.96
LD190910K	12 Leye	244	24°47′20.47”N, 106°34′30.28”E, elev 1130 m	8/27/2019	41.60	25.84
LD190910L	13 Leye	116	24°47′3.38”N, 106°34′35.56”E, elev 1136 m	8/27/2019	25.93	7.71
LD190910M	14 Leye	92	24°47′2.77”N, 106°34′35.08”E, elev 1142 m	8/27/2019	49.36	15.80
LD190910N	15 Leye	265	24°38′56.00”N, 106°39′0.70”E, elev 883 m	8/27/2019	45.87	20.58
LD190910O	16 Wangmo	120	25°14′59.65”N, 106°28′7.61”E, elev 717 m	8/31/2019	60.50	13.59
LD190910P	17 Wangmo	106	25°14′56.69”N, 106°28′8.62”E, elev 731 m	8/31/2019	38.10	20.29
LD190910Q	18 Wangmo	110	25°14′56.11”N, 106°28′9.10”E, elev 739 m	8/31/2019	52.40	14.56
LD190910R	19 Wangmo	70	25°14′40.42”N, 106°28′17.92”E, elev 761 m	8/31/2019	44.40	9.28
LD190910S	20 Wangmo	72	25°14′40.50”N, 106°28′18.04”E, elev 764 m	8/31/2019	49.60	15.73
LD190910T	21 Wangmo	85	25°14′40.90”N, 106°28′17.84”E, elev 765 m	8/31/2019	43.25	21.32
LD190910U	22 Wangmo	107	25°14′40.89”N, 106°28′17.08”E, elev 757 m	8/31/2019	47.60	21.32
LD190910V	23 Wangmo	145	25°13′48.24”N, 106° 7′44.64”E, elev 991 m	8/31/2019	61.40	17.51
LD190910W	24 Wangmo	80	25°13′47.85”N, 106° 7′44.85”E, elev 993 m	8/31/2019	36.65	14.05
LD190910X	25 Wangmo	105	25°13′50.79”N, 106° 7′44.79”E, elev 992 m	8/31/2019	3.96	15.04

dbh = Diameter at breast height, by convention measured at 1.3 m from the ground.

**Table 2:** Percentage compositions of the major components in *Liquidambar formosana* oleoresin essential oils.

Compound	Group #1	Group #2	Group #3	Group #4	Overall
	Average (Range)	Average (Range)	Average (Range)	Average (Range)	Average (Range)
(*E*)-Caryophyllene	14.2 (8.9–18.5)	26.0 (19.9–34.8)	5.0 (3.3–6.5)	49.0 (42.0–64.4)	23.5 (3.3–64.4)
α-Pinene	23.4 (18.7–27.8)	19.3 (15.3–25.3)	31.7 (29.1–34.5)	6.3 (0.6–11.8)	20.0 (0.6–34.5)
β-Pinene	16.9 (12.0–20.7)	14.2 (10.9–18.7)	23.3 (20.6–26.0)	5.4 (0.6–9.2)	14.8 (0.6–26.0)
Camphene	12.3 (9.2–15.3)	8.6 (6.0–10.9)	14.5 (11.0–17.3)	2.7 (0.3–5.2)	9.6 (0.3–17.3)
Limonene	5.8 (3.9–7.9)	3.6 (2.5–5.6)	6.7 (6.4–7.3)	0.9 (0.2–1.9)	4.3 (0.2–7.9)
Germacrene D	2.2 (0.0–6.5)	2.9 (0.0–9.9)	0.9 (0.2–1.8)	2.3 (0.2–5.5)	2.2 (0.0–9.9)
Camphor	3.3 (0.3–7.9)	1.6 (0.2–2.8)	1.2 (0.0–3.6)	1.0 (0.0–2.6)	1.9 (0.0–7.9)
β-Copaene	0.9 (0.3–1.5)	2.1 (0.4–5.3)	0.3 (0.1–0.6)	3.7 (2.7–4.9)	1.8 (0.1–5.3)
Bornyl acetate	1.7 (0.0–8.7)	1.3 (0.2–6.1)	3.1 (1.0–6.3)	1.3 (0.2–3.0)	1.7 (0.0–8.7)
α-Muurolol	1.1 (0.3–2.0)	1.8 (0.4–2.9)	0.3 (0.1–0.6)	3.7 (2.5–5.7)	1.7 (0.1–5.7)
*p*-Cymene	3.7 (0.1–13.6)	0.7 (0.1–1.9)	1.1 (0.2–3.8)	0.4 (0.0–0.7)	1.7 (0.0–13.6)
Sabinene	1.8 (0.4–3.1)	1.3 (0.4–1.9)	3.0 (2.4–3.6)	0.4 (0.0–1.1)	1.5 (0.0–3.6)
β-Cubebene	0.7 (0.2–1.2)	1.1 (0.4–1.8)	0.3 (0.1–0.6)	2.4 (0.1–3.4)	1.1 (0.1–3.4)
Caryophyllene oxide	0.8 (0.3–2.5)	1.0 (0.5–1.5)	0.3 (0.2–0.5)	1.6 (0.7–4.4)	0.9 (0.2–4.4)
α-Muurolene	0.4 (0.1–0.6)	0.9 (0.2–2.1)	0.1 (0.0–0.2)	1.9 (1.4–2.4)	0.8 (0.0–2.4)
Myrcene	0.7 (0.0–1.6)	0.4 (0.1–1.3)	2.2 (0.1–5.1)	0.4 (0.1–1.3)	0.8 (0.0–5.1)
(2*E*,4*E*)-3,7-Dimethylocta-2,4-diene	1.3 (0.3–3.9)	0.5 (0.1–1.0)	0.5 (0.1–0.9)	0.3 (0.0–0.6)	0.7 (0.0–3.9)
δ-Cadinene	0.3 (0.1–0.5)	0.7 (0.3–1.9)	0.1 (0.1–0.1)	1.5 (0.7–2.2)	0.7 (0.1–2.2)
γ-Muurolene	0.3 (0.1–0.5)	0.7 (0.2–1.7)	0.1 (0.0–0.2)	1.5 (1.0–1.9)	0.6 (0.0–1.9)
α-Humulene	0.3 (0.2–0.4)	0.7 (0.5–0.9)	0.1 (0.1–0.1)	1.4 (1.1–2.0)	0.6 (0.1–2.0)
Cubebol	0.3 (0.1–0.7)	0.6 (0.1–1.3)	0.1 (0.0–0.2)	1.2 (0.7–2.1)	0.5 (0.0–2.1)
*trans*-β-Elemene	0.1 (0.0–0.1)	1.3 (0.0–5.3)	0.2 (0.0–0.9)	0.2 (0.0–0.3)	0.5 (0.0–5.3)
Bornyl cinnamate	0.5 (0.1–1.2)	0.5 (0.1–0.8)	0.5 (0.3–0.8)	0.4 (0.2–1.0)	0.5 (0.1–1.2)
Borneol	0.4 (0.1–0.8)	0.6 (0.3–1.0)	0.5 (0.2–0.7)	0.4 (0.0–0.8)	0.5 (0.0–1.0)
α-Cubebene	0.1 (0.0–0.3)	0.6 (0.1–2.5)	0.0 (0.0–0.1)	0.8 (0.4–1.4)	0.4 (0.0–2.5)

**Table 3:** Antibacterial activities, MIC ^a^ (μg/mL), of oleoresin essential oils of *Liquidambar formosana* and major essential oil components.

Essential Oil Sample	Gram-Positive Bacteria					Gram-Negative Bacteria	
	*Bacillus Cereus*	*Cutibacterium Acnes* ^b^	*Staphylococcus Aureus*	*Staphylococcus Epidermidis*	*Streptococcus Pyogenes*	*Pseudomonas Aeruginosa*	*Serratia Marcescens*
LD190910C	156	156	156	78	156	313	625
LD190910D	156	156	156	78	156	313	625
LD190910E	156	156	156	78	156	313	625
LD190910F	156	156	156	78	156	313	625
LD190910G	156	156	156	78	156	313	625
LD190910H	156	156	156	78	156	313	625
LD190910I	156	156	156	78	156	313	625
LD190910J	156	156	156	78	156	313	625
LD190910K	156	156	156	78	156	313	625
LD190910L	156	156	156	78	156	313	625
LD190910M	156	156	156	78	156	313	625
LD190910N	156	156	156	78	156	313	625
LD190910O	156	156	156	78	156	313	625
LD190910P	156	156	156	78	156	313	625
LD190910Q	156	156	156	78	156	313	625
LD190910R	156	156	156	78	156	313	625
LD190910S	156	156	156	78	156	313	625
LD190910T	156	156	156	78	156	313	625
LD190910U	156	78	156	78	156	313	625
LD190910V	156	156	156	78	156	313	625
LD190910W	156	156	156	78	156	313	625
LD190910X	156	156	156	78	156	313	625
Re190401A	156	156	156	78	156	313	625
Re190401D	78	156	156	78	156	313	625
Re190401E	156	156	156	78	156	313	625
Pure Compounds							
(*E*)-Caryophyllene	313	625	313	313	313	313	313
(+)-α-Pinene	313	625	625	313	625	313	313
(–)-α-Pinene	313	625	313	313	313	313	313
(–)-β-Pinene	313	313	156	313	625	313	313
Camphene	313	625	313	313	313	313	313
(+)-Limonene	313	625	313	313	313	313	625
(–)-Limonene	313	39	313	78	625	313	313
Gentamicin	<19.5	<19.5	<19.5	<19.5	<19.5	<19.5	<19.5

^a^ Minimum inhibitory concentration. ^b^ Formerly *Propionibacterium acnes*.

**Table 4:** Antifungal activities, MIC ^a^ (μg/mL), of oleoresin essential oils of *Liquidambar formosana* and major essential oil components.

Essential Oil Sample	Molds						Yeast
	*Aspergillus Fumigatus*	*Aspergillus Niger*	*Microsporum Canis*	*Microsporum Gypseum*	*Trichophyton Mentagrophytes*	*Trichophyton Rubrum*	*Candida Albicans*
LD190910C	156	313	313	313	156	313	313
LD190910D	156	313	156	313	156	313	313
LD190910E	156	156	156	313	156	313	313
LD190910F	156	313	313	313	156	313	313
LD190910G	156	313	313	313	156	313	313
LD190910H	156	78	313	313	156	313	313
LD190910I	156	313	313	313	156	313	313
LD190910J	156	156	313	313	156	313	313
LD190910K	156	156	313	313	156	313	313
LD190910L	156	313	313	313	156	313	313
LD190910M	156	156	313	313	156	313	313
LD190910N	156	78	156	313	156	313	313
LD190910O	156	78	156	313	156	313	313
LD190910P	156	156	156	313	156	313	313
LD190910Q	156	313	156	313	156	313	313
LD190910R	156	313	156	313	156	313	313
LD190910S	156	313	156	313	156	313	313
LD190910T	156	313	156	313	156	313	313
LD190910U	156	156	156	313	156	313	313
LD190910V	156	156	156	313	156	313	313
LD190910W	156	313	156	313	156	313	313
LD190910X	156	313	156	313	156	313	313
Re190401A	156	313	156	313	156	313	313
Re190401D	156	313	156	313	156	313	313
Re190401E	156	313	156	313	156	313	313
Pure Compounds							
(*E*)-Caryophyllene	156	1250	313	313	625	313	156
(+)-α-Pinene	156	78	313	156	156	313	156
(–)-α-Pinene	313	156	313	313	313	313	156
(–)-β-Pinene	156	78	313	313	156	313	156
Camphene	313	156	313	313	625	313	156
(+)-Limonene	156	156	313	313	313	313	156
(–)-Limonene	156	156	313	156	156	313	156
Amphotericin B	<19.5	<19.5	<19.5	<19.5	<19.5	<19.5	<19.5

^a^ Minimum inhibitory concentration.

## Supplementary Materials

The following are available online at https://www.mdpi.com/2223-7747/9/7/822/s1, Table S1: Chemical compositions of *Liquidambar formosana* resin essential oils from southern China, Table S2: Enantiomeric distribution, (+)-enantiomer%: (–)-enantiomer%, of monoterpenoids in *Liquidambar formosana* resin essential oils.
