# Supplementary material for: The Essential Oil Composition and Antimicrobial Activity of Liquidambar formosana Oleoresin
Source: Plants (Basel). 2020 Jun 30;9(7):822. doi: 10.3390/plants9070822 (PMC7412184; doi:10.3390/plants9070822)
Supplement: Supplementary file 1 [file plants-09-00822-s001.pdf]

Table S1. Chemical compositions (%) of *Liquidambar formosana* oleoresin essential oils from southern China.

RI_{calc}	RI_{db}	Compound	RE190401A	RE190401D	RE190401E	LD190910C	LD190910D	LD190910E	LD190910F
763	763	Toluene	0.00	0.00	0.00	0.00	0.00	0.00	0.00
863	857	Ethylbenzene	0.09	0.00	0.00	0.00	0.00	0.00	0.00
893	891	Styrene	0.17	0.32	0.04	0.00	0.00	0.00	0.00
900	893	2-Bornene	0.00	0.00	0.00	0.00	0.00	0.00	0.00
908	910	(*E*)-2,6-Dimethyl-4-octene	0.03	0.00	0.00	0.00	0.00	0.00	0.00
920	919	5,5-Dimethyl-1-vinylbicyclo[2.1.1]hexane	0.00	0.00	0.00	0.00	0.00	0.00	0.00
923	922	Tricyclene	0.39	0.14	0.19	0.27	0.37	0.28	0.34
929	927	α-Thujene	0.32	0.30	0.28	0.25	0.53	0.52	0.70
**938**	**933**	**α-Pinene**	**22.24**	**15.52**	**19.97**	**18.75**	**34.54**	**25.42**	**33.06**
945	948	β-Citronellene	0.00	0.00	0.00	0.20	0.00	0.00	0.00
948	947	Allylbenzene	0.03	0.02	0.04	0.51	0.18	0.08	0.02
951	948	α-Fenchene	0.05	0.00	0.00	0.00	0.00	0.00	0.00
**953**	**953**	**Camphene**	**15.32**	**5.99**	**8.45**	**12.78**	**15.82**	**11.45**	**13.90**
956	953	Thuja-2,4(10)-diene	0.02	0.01	0.01	0.00	0.00	0.01	0.01
964	964	Benzaldehyde	0.00	0.00	0.00	0.00	0.00	0.00	0.00
966	964	(*Z*)-3,7-Dimethyl-2-octene	0.24	0.00	0.02	2.16	0.12	0.05	0.01
**973**	**972**	**Sabinene**	**1.42**	**0.84**	**1.38**	**0.91**	**2.39**	**2.53**	**3.56**
**979**	**978**	**β-Pinene**	**14.28**	**11.57**	**14.49**	**14.38**	**20.59**	**19.98**	**23.76**
982	988	α-Methylstyrene	0.00	0.00	0.00	0.00	0.00	0.00	0.00
**987**	**991**	**Myrcene**	**0.04**	**0.47**	**0.16**	**0.06**	**0.15**	**1.13**	**1.83**
989	990	2,3-Dehydro-1,8-cineole	0.24	0.02	0.01	0.00	0.00	0.00	0.00
991	990	*o*-Methylstyrene	0.00	0.00	0.00	0.07	0.02	0.00	0.00
999	997	*p*-Menth-2-ene	0.44	0.01	0.01	1.75	0.36	0.13	0.01
1002	1004	*p*-Menth-1(7),8-diene	0.00	0.00	0.00	0.00	0.00	0.00	0.00
1005	1007	α-Phellandrene	0.00	0.03	0.03	0.05	0.04	0.04	0.07
1007	1008	δ-3-Carene	0.00	0.00	0.00	0.00	0.02	0.01	0.01
1015	1018	α-Terpinene	0.05	0.05	0.05	0.77	0.19	0.08	0.07
**1021**	**1022**	**(2*E*,4*E*)-3,7-Dimethylocta-2,4-diene**	**0.79**	**0.10**	**0.34**	**3.90**	**0.91**	**0.64**	**0.85**
**1023**	**1025**	***p*-Cymene**	**4.04**	**0.06**	**0.19**	**13.55**	**3.76**	**1.24**	**0.19**
1026	1028	Indane	0.00	0.00	0.00	0.22	0.10	0.00	0.00
**1028**	**1030**	**Limonene**	**4.81**	**3.27**	**2.86**	**5.40**	**6.36**	**5.61**	**6.42**
1030	1031	β-Phellandrene	0.05	0.19	0.07	0.05	0.05	0.43	0.57
1031	1032	1,8-Cineole	0.09	0.03	0.05	0.09	0.15	0.10	0.23
1034	1034	(*Z*)-β-Ocimene	0.02	0.01	0.01	0.01	0.02	0.02	0.04
1044	1046	(*E*)-β-Ocimene	0.00	0.02	0.01	0.01	0.01	0.02	0.12
1057	1058	γ-Terpinene	0.21	0.08	0.07	1.04	0.58	0.19	0.13
1060	1064	Benzenemethanol	0.00	0.03	0.00	0.00	0.00	0.00	0.00
1066	1068	Acetophenone	0.15	0.38	0.15	0.18	0.00	0.13	0.02
1069	1069	*cis*-Sabinene hydrate	0.12	0.02	0.02	0.03	0.05	0.05	0.12
1085	1086	Terpinolene	0.08	0.08	0.09	0.23	0.15	0.13	0.24
1089	1090	Fenchone	0.02	0.00	0.01	0.00	0.00	0.00	0.00
1091	1093	*p*-Cymenene	0.00	0.00	0.00	0.00	0.00	0.00	0.00
1099	1098	Perillene	0.00	0.00	0.00	0.01	0.00	0.00	0.00
1099	1101	α-Pinene oxide	0.00	0.00	0.00	0.00	0.00	0.00	0.00
1100	1101	Linalool	0.00	0.04	0.02	0.01	0.02	0.08	0.09
1101	1101	*trans*-Sabinene hydrate	0.02	0.01	0.01	0.01	0.01	0.01	0.02
1121	1121	*exo*-Fenchol (= β-Fenchol)	0.02	0.00	0.01	0.00	0.00	0.00	0.00
1123	1122	Chrystanthenone	0.02	0.00	0.00	0.00	0.00	0.00	0.00
1123	1122	*trans*-*p*-Mentha-2,8-dien-1-ol	0.00	0.00	0.00	0.00	0.00	0.00	0.00
1125	1224	*p*-Menth-1-en-9-al	0.00	0.00	0.00	0.00	0.00	0.00	0.00
1126	1124	*cis*-*p*-Menth-2-en-1-ol	0.00	0.00	0.00	0.00	0.01	0.00	0.00
1128	1127	α-Campholenal	0.00	0.00	0.00	0.00	0.00	0.00	0.00
1134	1134	*cis*-Limonene oxide	0.00	0.00	0.00	0.00	0.00	0.00	0.00
1143	1141	*trans*-Pinocarveol	0.00	0.00	0.00	0.00	0.01	0.02	0.01
1144	1141	*cis*-Verbenol	0.00	0.00	0.00	0.00	0.03	0.00	0.00
1145	1142	*trans*-*p*-Menth-2-en-1-ol	0.00	0.00	0.00	0.00	0.00	0.00	0.00

RIcalc	RIdb	Compound	RE190401A	RE190401D	RE190401E	LD190910C	LD190910D	LD190910E	LD190910F
1145	1143	*trans*-Dihydro-β-terpineol	0.00	0.00	0.00	0.00	0.00	0.00	0.00
1148	1146	*trans*-Verbenol	0.00	0.01	0.02	0.00	0.08	0.03	0.01
**1150**	**1149**	**Camphor**	**7.87**	**1.62**	**2.80**	**4.15**	**3.60**	**0.72**	**0.55**
1151	1149	*trans*-Dihydro-α-terpineol	0.00	0.00	0.00	0.00	0.00	0.00	0.00
1152	1150	α-Phellandren-8-ol	0.00	0.00	0.00	0.00	0.01	0.00	0.00
1158	1156	Camphene hydrate	0.04	0.00	0.00	0.07	0.01	0.01	0.00
1159	1157	Sabina ketone	0.00	0.00	0.00	0.00	0.00	0.00	0.00
1160	1158	Menthone	0.00	0.00	0.00	0.00	0.00	0.00	0.00
1163	1160	*trans*-Pinocamphone	0.00	0.00	0.00	0.01	0.02	0.01	0.00
1165	1164	Pinocarvone	0.03	0.03	0.02	0.02	0.01	0.03	0.01
1167	1164	*cis*-Dihydro-α-terpineol	0.00	0.00	0.00	0.00	0.00	0.00	0.00
1167	1165	Isoborneol	0.00	0.00	0.00	0.00	0.01	0.00	0.00
1170	1171	4-Ethylphenol	0.00	0.00	0.00	0.00	0.00	0.00	0.00
**1175**	**1173**	**Borneol**	**0.13**	**0.79**	**1.04**	**0.14**	**0.65**	**0.29**	**0.56**
1179	1176	*cis*-Pinocamphone	0.19	0.00	0.00	0.02	0.02	0.01	0.01
1181	1179	*iso*-Menthol	0.00	0.00	0.00	0.02	0.00	0.00	0.00
1181	1179	2-Isopropenyl-5-methyl-4-hexenal	0.00	0.00	0.00	0.00	0.00	0.00	0.00
1184	1180	Terpinen-4-ol	0.38	0.15	0.11	0.46	0.27	0.19	0.15
1187	1183	Myrtanal	0.00	0.00	0.00	0.00	0.00	0.00	0.00
1190	1187	Cryptone	0.00	0.00	0.00	0.00	0.00	0.01	0.00
1190	1189	*p*-Cymen-8-ol	0.02	0.00	0.00	0.00	0.00	0.01	0.00
1198	1202	Myrtenol	0.00	0.00	0.00	0.00	0.00	0.00	0.00
1199	1195	α-Terpineol	0.22	0.44	0.29	0.18	0.32	0.44	0.42
1202	1198	(*Z*)-Dihydrocarvone	0.04	0.00	0.00	0.00	0.00	0.00	0.00
1202	1201	Estragole (= Methyl chavicol)	0.00	0.00	0.00	0.00	0.00	0.00	0.03
1211	1208	Verbenone	0.21	0.01	0.05	0.04	0.05	0.02	0.01
1234	1235	Hydrocinnamic alcohol (= Benzenepropanol)	0.00	0.57	0.10	0.00	0.00	0.00	0.00
1235	1232	*cis*-Carveol	0.00	0.00	0.00	0.00	0.00	0.00	0.00
1247	1246	Carvone	0.07	0.00	0.00	0.00	0.00	0.00	0.00
1250	1246	*trans*-Shisool	0.00	0.00	0.00	0.00	0.00	0.00	0.00
1274	1276	2,3-Pinanediol	0.00	0.00	0.00	0.00	0.00	0.00	0.00
1278	1273	(*E*)-Cinnamaldehyde	0.00	0.14	0.01	0.00	0.00	0.04	0.01
1280	1278	Perilla aldehyde	0.00	0.00	0.02	0.00	0.00	0.00	0.00
1283	1278	*cis*-Verbenyl acetate	0.00	0.00	0.00	0.00	0.00	0.00	0.00
**1286**	**1285**	**Bornyl acetate**	**0.09**	**0.37**	**0.86**	**0.04**	**1.03**	**0.12**	**1.31**
1287	1287	Isobornyl acetate	0.00	0.00	0.00	0.00	0.00	0.00	0.00
1300	1297	*p*-Menth-1-en-9-ol	0.00	0.00	0.00	0.00	0.04	0.00	0.00
1300	1299	Perilla alcohol	0.04	0.02	0.04	0.00	0.00	0.00	0.00
1300	1300	Carvacrol	0.03	0.00	0.00	0.00	0.00	0.00	0.00
1309	1309	(*E*)-Cinnamyl alcohol	0.00	0.47	0.05	0.00	0.00	0.00	0.00
1331	1334	Bicycloelemene	0.00	0.00	0.02	0.00	0.00	0.00	0.00
1334	1335	δ-Elemene	0.00	0.00	0.08	0.00	0.00	0.00	0.00
**1349**	**1349**	**α-Cubebene**	**0.14**	**2.51**	**0.50**	**0.03**	**0.02**	**0.13**	**0.04**
1352	1352	Ethyl hydrocinnamate	0.00	0.00	0.00	0.00	0.00	0.00	0.00
1371	1367	Cyclosativene	0.12	0.57	0.31	0.04	0.01	0.10	0.05
1377	1375	α-Copaene	0.05	0.36	0.17	0.04	0.18	0.57	0.11
1380	1384	Daucene	0.00	0.01	0.00	0.00	0.00	0.00	0.00
1383	1383	*cis*-β-Elemene	0.12	0.00	0.24	0.00	0.00	0.00	0.00
1383	1385	Isopropyl 3-phenylpropanoate	0.00	0.00	0.00	0.00	0.00	0.00	0.00
1385	1382	β-Bourbonene	0.01	0.02	0.04	0.00	0.01	0.02	0.01
**1389**	**1392**	**β-Cubebene**	**0.46**	**1.22**	**0.97**	**0.20**	**0.08**	**1.08**	**0.59**
**1391**	**1390**	***trans*-β-Elemene**	**0.00**	**0.08**	**5.28**	**0.02**	**0.01**	**0.10**	**0.03**
1394	1394	Sativene	0.00	0.02	0.01	0.00	0.00	0.00	0.00
1406	1405	(*Z*)-Caryophyllene	0.05	0.01	0.01	0.02	0.01	0.00	0.00
**1423**	**1424**	**(*E*)-Caryophyllene**	**14.17**	**29.84**	**19.91**	**14.29**	**4.14**	**14.80**	**5.97**
1423	1428	β-Duprezianene	0.00	0.00	0.00	0.00	0.00	0.00	0.01
1430	1432	γ-Elemene	0.00	0.00	0.00	0.00	0.00	0.00	0.00
**1431**	**1433**	**β-Copaene**	**0.84**	**5.32**	**2.14**	**0.31**	**0.05**	**0.99**	**0.52**
1433	1432	*trans*-α-Bergamotene	0.00	0.00	0.00	0.00	0.00	0.00	0.00
1436	1436	α-Guaiene	0.00	0.00	2.59	0.00	0.00	0.00	0.00

RIcalc	RIdb	Compound	RE190401A	RE190401D	RE190401E	LD190910C	LD190910D	LD190910E	LD190910F
1439	1442	6,9-Guaiadiene	0.00	0.01	0.01	0.00	0.00	0.00	0.00
1440	1439	(*Z*)-β-Farnesene	0.00	0.00	0.00	0.00	0.00	0.00	0.00
1444	1445	Selina-5,11-diene	0.00	0.00	0.01	0.00	0.00	0.00	0.00
1445	1447	*iso*-Germacrene D	0.00	0.00	0.01	0.00	0.00	0.00	0.00
1446	1446	*cis*-Muurola-3,5-diene	0.00	0.05	0.01	0.00	0.00	0.00	0.00
1450	1452	*trans*-Muurola-3,5-diene	0.02	0.33	0.07	0.00	0.00	0.02	0.01
1451	1452	(*E*)-β-Farnesene	0.00	0.02	0.00	0.00	0.00	0.00	0.00
1452	1453	ε-Muurolene	0.00	0.00	0.00	0.00	0.00	0.03	0.00
1452	1455	Sesquisabinene	0.00	0.00	0.00	0.00	0.00	0.00	0.00
1453	1455	Valerena-4,7(11)-diene	0.00	0.00	0.00	0.00	0.00	0.00	0.00
**1456**	**1454**	**α-Humulene**	**0.25**	**0.68**	**0.71**	**0.27**	**0.08**	**0.34**	**0.12**
1459	1461	*cis*-Cadina-1(6),4-diene	0.00	0.00	0.00	0.00	0.00	0.00	0.00
1461	1464	9-*epi*-(*E*)-Caryophyllene	0.00	0.00	0.00	0.00	0.00	0.02	0.00
1463	1463	*cis*-Muurola-4(14),5-diene	0.00	0.08	0.02	0.00	0.00	0.04	0.00
1470	1471	4,5-di-*epi*-Aristolochene	0.00	0.00	0.01	0.00	0.00	0.00	0.00
1472	1472	*trans*-Cadina-1(6),4-diene	0.01	0.33	0.05	0.01	0.00	0.02	0.00
1473	1475	Selina-4,11-diene	0.00	0.00	0.12	0.00	0.00	0.00	0.00
**1475**	**1478**	**γ-Muurolene**	**0.27**	**1.72**	**0.67**	**0.09**	**0.02**	**0.35**	**0.15**
1478	1480	*cis*-4,10-Epoxyamorphane	0.21	0.72	0.18	0.03	0.01	0.10	0.05
1478	1483	α-Amorphene	0.00	0.00	0.04	0.00	0.00	0.00	0.00
**1482**	**1480**	**Germacrene D**	**0.01**	**0.47**	**0.68**	**0.12**	**0.42**	**5.31**	**1.08**
1487	1488	δ-Selinene	0.00	0.00	0.00	0.00	0.00	0.00	0.00
1488	1491	Eremophilene	0.00	0.00	0.02	0.00	0.00	0.00	0.00
1490	1487	β-Selinene	0.00	0.04	0.68	0.00	0.00	0.00	0.00
1493	1490	γ-Amorphene	0.03	0.53	0.11	0.01	0.00	0.08	0.02
1496	1497	α-Selinene	0.00	0.00	0.74	0.00	0.00	0.00	0.00
1496	1498	*epi*-Cubebol	0.16	0.60	0.00	0.04	0.02	0.14	0.04
1497	1498	Bicyclogermacrene	0.00	0.00	0.00	0.00	0.00	0.00	0.00
**1499**	**1497**	**α-Muurolene**	**0.33**	**2.13**	**0.81**	**0.11**	**0.02**	**0.39**	**0.18**
1502	1497	Valencene	0.00	0.00	0.00	0.00	0.00	0.00	0.00
1502	1504	*iso*-Daucene	0.00	0.09	0.00	0.05	0.00	0.04	0.00
1503	1503	(*E,E*)-α-Farnesene	0.00	0.00	0.00	0.00	0.00	0.02	0.03
1503	1506	δ-Amorphene	0.00	0.07	0.00	0.00	0.00	0.00	0.00
1505	1505	α-Bulnesene	0.00	0.00	2.25	0.00	0.00	0.00	0.00
1508	1508	β-Bisabolene	0.00	0.00	0.00	0.00	0.00	0.00	0.00
1509	1511	Germacrene A	0.00	0.00	0.00	0.00	0.00	0.00	0.00
1514	1512	γ-Cadinene	0.00	0.00	0.00	0.00	0.00	0.04	0.00
**1516**	**1519**	**Cubebol**	**0.71**	**1.00**	**0.41**	**0.08**	**0.02**	**0.25**	**0.14**
**1518**	**1518**	**δ-Cadinene**	**0.15**	**1.88**	**0.50**	**0.08**	**0.08**	**0.42**	**0.10**
1521	1519	*trans*-Calamenene	0.03	0.00	0.00	0.00	0.00	0.00	0.00
1523	1521	Zonarene	0.01	0.19	0.05	0.00	0.00	0.00	0.00
1525	1524	β-Sesquiphellandrene	0.00	0.00	0.00	0.00	0.00	0.00	0.00
1533	1536	*trans*-Cadina-1,4-diene	0.02	0.25	0.05	0.00	0.00	0.00	0.01
1537	1538	α-Cadinene	0.00	0.00	0.00	0.00	0.00	0.00	0.00
1541	1541	α-Calacorene	0.00	0.00	0.00	0.00	0.00	0.00	0.00
1548	1546	α-Elemol	0.00	0.00	0.00	0.00	0.00	0.00	0.01
1559	1557	Germacrene B	0.00	0.05	0.15	0.00	0.00	0.00	0.00
1570	1566	1,5-Epoxysalvial-4(14)ene	0.00	0.00	0.00	0.00	0.00	0.00	0.00
1577	1576	Spathulenol	0.00	0.00	0.00	0.00	0.02	0.00	0.00
1578	1575	Germacra-1(10),5-dien-4β-ol	0.00	0.00	0.00	0.00	0.00	0.00	0.01
**1584**	**1587**	**Caryophyllene oxide**	**2.50**	**0.49**	**0.74**	**0.57**	**0.46**	**0.68**	**0.15**
1596	1596	Salvial-4(14)-en-1-one	0.00	0.00	0.00	0.00	0.01	0.00	0.00
1600	1600	Hexadecane	0.00	0.00	0.00	0.00	0.00	0.00	0.00
1615	1613	Humulene epoxide II	0.10	0.00	0.00	0.00	0.01	0.00	0.00
1628	1623	Humulane-1,6-dien-3-ol	0.00	0.00	0.00	0.00	0.04	0.20	0.02
1628	1627	Germacra-1(10),5-dien-4α-ol	0.00	0.04	0.02	0.00	0.00	0.00	0.00
1634	1631	1-*epi*-Cubenol	0.00	0.17	0.09	0.00	0.00	0.00	0.00
1634	---	Unidentified	0.30	0.49	0.24	0.04	0.01	0.00	0.00
1635	1629	*iso*-Spathulenol	0.00	0.00	0.00	0.00	0.00	0.00	0.00
1640	1633	γ-Eudesmol	0.00	0.00	0.00	0.00	0.00	0.00	0.00
1640	1642	Caryophylla-4(12),8(13)-dien-5α-ol	0.06	0.00	0.00	0.00	0.00	0.00	0.00

RIcalc	RIdb	Compound	RE190401A	RE190401D	RE190401E	LD190910C	LD190910D	LD190910E	LD190910F
1641	1644	Caryophylla-4(12),8(13)-dien-5β-ol	0.00	0.00	0.00	0.00	0.00	0.00	0.00
1642	1638	(2*S*,5*E*)-Caryophyll-5-en-12-al	0.05	0.00	0.00	0.00	0.00	0.00	0.00
1647	1643	Cubenol	0.00	0.18	0.06	0.00	0.00	0.00	0.00
**1650**	**1651**	***α*-Muurolol**	**2.01**	**2.92**	**1.65**	**0.27**	**0.06**	**0.74**	**0.41**
1658	1655	*α*-Eudesmol	0.00	0.00	0.00	0.00	0.00	0.00	0.00
1658	1655	*α*-Cadinol	0.00	0.00	0.00	0.00	0.00	0.00	0.00
1658	1656	β-Eudesmol	0.03	0.00	0.03	0.00	0.00	0.00	0.00
1660	1660	Selin-11-en-4*α*-ol	0.04	0.00	1.67	0.00	0.00	0.00	0.00
1671	1666	14-Hydroxy-9-*epi*-(*E*)-caryophyllene	0.74	0.00	0.00	0.00	0.00	0.00	0.00
1681	1676	Bornyl 8-hydroxyisobutanoate	0.00	0.00	0.00	0.00	0.00	0.00	0.00
2052	2050	Bornyl hydrocinnamate	0.00	0.00	0.02	0.00	0.02	0.00	0.00
2151	2152	3-Phenylpropyl 3-phenylpropanoate	0.00	0.00	0.02	0.00	0.00	0.00	0.00
**2224**	**2223**	**Bornyl cinnamate**	**0.35**	**0.14**	**0.27**	**0.36**	**0.45**	**0.44**	**0.54**
2318	2321	3-Phenylpropyl cinnamate	0.00	0.04	0.00	0.00	0.00	0.00	0.00
2434	2432	Phenylallyl cinnamate	0.00	0.03	0.00	0.00	0.00	0.02	0.00
		Monoterpene hydrocarbons	64.79	38.75	48.68	76.52	86.95	69.89	85.89
		Oxygenated monoterpenoids	9.87	3.57	5.37	5.29	6.41	2.15	3.50
		Sesquiterpene hydrocarbons	17.08	48.86	40.05	15.69	5.13	24.91	9.02
		Oxygenated sesquiterpenoids	6.60	6.11	4.86	0.99	0.65	2.10	0.83
		Benzenoid aromatics	0.78	2.13	0.69	1.34	0.77	0.72	0.62
		Others	0.00	0.00	0.00	0.00	0.00	0.00	0.00
		Total identified	99.12	99.43	99.65	99.84	99.91	99.78	99.86

RIcalc: Retention indices determined with respect to a homologous series of *n*-alkanes on a ZB-5ms column. RIdb: Retention indices from the databases. Compounds in **boldface** were used in the multivariate (HCA and PCA) analyses.

Table S1. Continued.

RIcalc	RIdb	Compound	LD190910G	LD190910H	LD190910I	LD190910J	LD190910K	LD190910L	LD190910M
763	763	Toluene	0.00	0.00	0.00	0.00	0.00	0.00	0.00
863	857	Ethylbenzene	0.00	0.00	0.00	0.00	0.00	0.00	0.05
893	891	Styrene	0.00	0.00	0.00	0.06	0.00	0.00	0.03
900	893	2-Bornene	0.00	0.00	0.00	0.00	0.00	0.00	0.00
908	910	(*E*)-2,6-Dimethyl-4-octene	0.00	0.00	0.00	0.00	0.00	0.00	0.00
920	919	5,5-Dimethyl-1-vinylbicyclo[2.1.1]hexane	0.00	0.00	0.00	0.00	0.00	0.00	0.01
923	922	Tricyclene	0.30	0.23	0.34	0.27	0.42	0.30	0.20
929	927	*α*-Thujene	0.41	0.33	0.45	0.44	0.53	0.42	0.24
**938**	**933**	***α*-Pinene**	**27.28**	**22.70**	**27.80**	**25.35**	**30.06**	**23.74**	**21.18**
945	948	β-Citronellene	0.00	0.00	0.00	0.00	0.00	0.00	0.02
948	947	Allylbenzene	0.00	0.00	0.01	0.32	0.01	0.09	0.47
951	948	*α*-Fenchene	0.00	0.00	0.00	0.00	0.00	0.00	0.00
**953**	**953**	**Camphene**	**11.90**	**9.29**	**13.70**	**10.93**	**17.32**	**13.62**	**9.18**
956	953	Thuja-2,4(10)-diene	0.01	0.01	0.01	0.02	0.02	0.03	0.00
964	964	Benzaldehyde	0.00	0.00	0.00	0.00	0.00	0.00	0.00
966	964	(*Z*)-3,7-Dimethyl-2-octene	0.01	0.00	0.00	0.09	0.01	0.12	2.27
**973**	**972**	**Sabinene**	**2.33**	**1.95**	**3.11**	**1.34**	**3.58**	**1.64**	**0.38**
**979**	**978**	**β-Pinene**	**19.12**	**18.67**	**20.75**	**17.17**	**22.68**	**18.67**	**12.03**
982	988	*α*-Methylstyrene	0.00	0.00	0.00	0.00	0.00	0.00	0.00
**987**	**991**	**Myrcene**	**1.44**	**1.33**	**1.64**	**0.23**	**1.89**	**0.04**	**0.11**
989	990	2,3-Dehydro-1,8-cineole	0.00	0.00	0.00	0.00	0.00	0.00	0.00
991	990	*o*-Methylstyrene	0.00	0.00	0.00	0.00	0.00	0.00	0.00
999	997	*p*-Menth-2-ene	0.00	0.00	0.00	0.12	0.02	0.84	2.88

RI$_{calc}$	RI$_{db}$	Compound	LD1909 10G	LD1909 10H	LD190 910I	LD190 910J	LD190 910K	LD190 910L	LD1909 10M
1002	1004	*p*-Menth-1(7),8-diene	0.00	0.00	0.00	0.00	0.00	0.01	0.00
1005	1007	α-Phellandrene	0.03	0.02	0.02	0.03	0.06	0.02	0.10
1007	1008	δ-3-Carene	0.01	0.01	0.02	0.01	0.02	0.01	0.02
1015	1018	α-Terpinene	0.03	0.02	0.02	0.12	0.07	0.16	0.43
**1021**	**1022**	**(2*E*,4*E*)-3,7-Dimethylocta-2,4-diene**	**0.32**	**0.18**	**0.34**	**0.51**	**0.17**	**0.29**	**3.91**
**1023**	**1025**	***p*-Cymene**	**0.05**	**0.05**	**0.08**	**1.45**	**0.36**	**2.81**	**7.83**
1026	1028	Indane	0.00	0.00	0.00	0.00	0.00	0.14	0.11
**1028**	**1030**	**Limonene**	**6.45**	**4.52**	**6.13**	**4.53**	**7.30**	**6.12**	**3.87**
1030	1031	β-Phellandrene	0.26	0.24	0.24	0.09	0.23	0.00	0.05
1031	1032	1,8-Cineole	0.12	0.08	0.10	0.12	0.13	0.20	0.06
1034	1034	(*Z*)-β-Ocimene	0.03	0.02	0.03	0.01	0.03	0.02	0.01
1044	1046	(*E*)-β-Ocimene	0.04	0.04	0.04	0.01	0.04	0.00	0.01
1057	1058	γ-Terpinene	0.06	0.04	0.04	0.48	0.07	0.48	0.45
1060	1064	Benzenemethanol	0.00	0.00	0.00	0.00	0.00	0.00	0.00
1066	1068	Acetophenone	0.03	0.00	0.03	0.06	0.00	0.04	0.01
1069	1069	*cis*-Sabinene hydrate	0.09	0.05	0.05	0.04	0.05	0.06	0.05
1085	1086	Terpinolene	0.16	0.11	0.11	0.10	0.28	0.18	0.46
1089	1090	Fenchone	0.00	0.00	0.00	0.01	0.00	0.02	0.01
1091	1093	*p*-Cymenene	0.00	0.00	0.00	0.00	0.00	0.00	0.00
1099	1098	Perillene	0.00	0.00	0.00	0.00	0.00	0.00	0.01
1099	1101	α-Pinene oxide	0.00	0.03	0.03	0.00	0.03	0.00	0.00
1100	1101	Linalool	0.04	0.01	0.05	0.01	0.03	0.06	0.02
1101	1101	*trans*-Sabinene hydrate	0.02	0.01	0.01	0.01	0.02	0.01	0.01
1121	1121	*exo*-Fenchol (= β-Fenchol)	0.00	0.00	0.00	0.01	0.00	0.02	0.01
1123	1122	Chrystanthenone	0.00	0.00	0.00	0.00	0.00	0.00	0.00
1123	1122	*trans*-*p*-Mentha-2,8-dien-1-ol	0.00	0.00	0.00	0.00	0.01	0.01	0.00
1125	1224	*p*-Menth-1-en-9-al	0.00	0.00	0.00	0.00	0.00	0.00	0.00
1126	1124	*cis*-*p*-Menth-2-en-1-ol	0.00	0.00	0.00	0.01	0.00	0.02	0.01
1128	1127	α-Campholenal	0.00	0.00	0.00	0.00	0.01	0.00	0.00
1134	1134	*cis*-Limonene oxide	0.00	0.00	0.00	0.00	0.01	0.00	0.00
1143	1141	*trans*-Pinocarveol	0.02	0.03	0.03	0.01	0.04	0.02	0.00
1144	1141	*cis*-Verbenol	0.00	0.00	0.00	0.00	0.00	0.04	0.00
1145	1142	*trans*-*p*-Menth-2-en-1-ol	0.00	0.00	0.00	0.01	0.00	0.00	0.01
1145	1143	*trans*-Dihydro-β-terpineol	0.00	0.00	0.00	0.00	0.00	0.00	0.01
1148	1146	*trans*-Verbenol	0.01	0.02	0.02	0.02	0.03	0.08	0.00
**1150**	**1149**	**Camphor**	**0.35**	**0.26**	**0.25**	**2.59**	**0.54**	**4.69**	**6.17**
1151	1149	*trans*-Dihydro-α-terpineol	0.00	0.00	0.00	0.00	0.00	0.31	0.00
1152	1150	α-Phellandren-8-ol	0.00	0.00	0.00	0.01	0.00	0.00	0.01
1158	1156	Camphene hydrate	0.00	0.00	0.00	0.01	0.00	0.03	0.02
1159	1157	Sabina ketone	0.00	0.00	0.00	0.00	0.00	0.01	0.00
1160	1158	Menthone	0.00	0.00	0.00	0.00	0.00	0.00	0.03
1163	1160	*trans*-Pinocamphone	0.00	0.00	0.00	0.02	0.01	0.02	0.03
1165	1164	Pinocarvone	0.01	0.02	0.02	0.04	0.02	0.03	0.00
1167	1164	*cis*-Dihydro-α-terpineol	0.00	0.00	0.00	0.00	0.00	0.01	0.00
1167	1165	Isoborneol	0.00	0.00	0.00	0.00	0.00	0.02	0.03
1170	1171	4-Ethylphenol	0.00	0.00	0.00	0.00	0.00	0.00	0.00
**1175**	**1173**	**Borneol**	**0.34**	**0.49**	**0.36**	**0.33**	**0.62**	**0.71**	**0.30**
1179	1176	*cis*-Pinocamphone	0.01	0.01	0.01	0.01	0.02	0.03	0.02
1181	1179	*iso*-Menthol	0.00	0.00	0.00	0.00	0.00	0.00	0.00
1181	1179	2-Isopropenyl-5-methyl-4-hexenal	0.00	0.00	0.00	0.01	0.00	0.00	0.00
1184	1180	Terpinen-4-ol	0.12	0.09	0.05	0.27	0.10	0.59	0.71
1187	1183	Myrtanal	0.00	0.01	0.01	0.01	0.01	0.00	0.00
1190	1187	Cryptone	0.00	0.00	0.00	0.00	0.00	0.00	0.00
1190	1189	*p*-Cymen-8-ol	0.00	0.01	0.01	0.01	0.01	0.01	0.00
1198	1202	Myrtenol	0.00	0.00	0.00	0.05	0.00	0.00	0.00
1199	1195	α-Terpineol	0.17	0.18	0.30	0.11	0.24	0.56	0.24
1202	1198	(Z)-Dihydrocarvone	0.00	0.00	0.00	0.00	0.00	0.00	0.00
1202	1201	Estragole (= Methyl chavicol)	0.03	0.01	0.02	0.06	0.03	0.10	0.00
1211	1208	Verbenone	0.01	0.01	0.02	0.05	0.02	0.12	0.02

RI_{calc}	RI_{db}	Compound	LD190910G	LD190910H	LD190910I	LD190910J	LD190910K	LD190910L	LD190910M
1234	1235	Hydrocinnamic alcohol (= Benzenepropanol)	0.00	0.00	0.00	0.02	0.00	0.08	0.00
1235	1232	*cis*-Carveol	0.00	0.00	0.00	0.00	0.00	0.03	0.00
1247	1246	Carvone	0.00	0.00	0.00	0.00	0.01	0.02	0.00
1250	1246	*trans*-Shisool	0.00	0.00	0.00	0.00	0.00	0.01	0.00
1274	1276	2,3-Pinanediol	0.00	0.01	0.00	0.00	0.00	0.00	0.00
1278	1273	(*E*)-Cinnamaldehyde	0.01	0.01	0.02	0.02	0.00	0.00	0.00
1280	1278	Perilla aldehyde	0.00	0.00	0.00	0.00	0.00	0.00	0.00
1283	1278	*cis*-Verbenyl acetate	0.00	0.00	0.00	0.00	0.00	0.00	0.00
**1286**	**1285**	**Bornyl acetate**	**1.45**	**2.05**	**0.68**	**0.16**	**3.87**	**2.06**	**0.69**
1287	1287	Isobornyl acetate	0.00	0.00	0.00	0.00	0.01	0.02	0.00
1300	1297	*p*-Menth-1-en-9-ol	0.00	0.00	0.00	0.00	0.00	0.00	0.00
1300	1299	Perilla alcohol	0.00	0.00	0.01	0.00	0.00	0.00	0.00
1300	1300	Carvacrol	0.00	0.00	0.00	0.00	0.00	0.00	0.00
1309	1309	(*E*)-Cinnamyl alcohol	0.00	0.00	0.00	0.00	0.00	0.00	0.00
1331	1334	Bicycloelemene	0.01	0.01	0.01	0.00	0.00	0.00	0.08
1334	1335	δ-Elemene	0.00	0.00	0.00	0.00	0.01	0.00	0.00
**1349**	**1349**	***α*-Cubebene**	**0.05**	**0.06**	**0.08**	**0.38**	**0.02**	**0.32**	**0.18**
1352	1352	Ethyl hydrocinnamate	0.00	0.00	0.00	0.00	0.00	0.00	0.00
1371	1367	Cyclosativene	0.06	0.04	0.07	0.10	0.03	0.16	0.14
1377	1375	*α*-Copaene	0.39	0.63	0.60	0.06	0.27	0.12	0.10
1380	1384	Daucene	0.01	0.01	0.01	0.00	0.00	0.00	0.00
1383	1383	*cis*-β-Elemene	0.01	0.01	0.01	0.00	0.04	0.00	0.00
1383	1385	Isopropyl 3-phenylpropanoate	0.00	0.00	0.00	0.00	0.00	0.01	0.00
1385	1382	β-Bourbonene	0.04	0.05	0.05	0.00	0.03	0.02	0.02
**1389**	**1392**	**β-Cubebene**	**0.76**	**0.61**	**0.82**	**0.44**	**0.32**	**0.64**	**0.57**
**1391**	**1390**	***trans*-β-Elemene**	**0.11**	**0.23**	**0.09**	**0.03**	**0.87**	**0.02**	**0.02**
1394	1394	Sativene	0.00	0.00	0.00	0.00	0.00	0.00	0.00
1406	1405	(*Z*)-Caryophyllene	0.00	0.00	0.00	0.06	0.00	0.04	0.02
**1423**	**1424**	**(*E*)-Caryophyllene**	**14.74**	**21.59**	**11.41**	**24.27**	**3.27**	**8.88**	**18.50**
1423	1428	β-Duprezianene	0.00	0.00	0.00	0.00	0.00	0.00	0.00
1430	1432	γ-Elemene	0.00	0.00	0.00	0.00	0.00	0.00	0.00
**1431**	**1433**	**β-Copaene**	**0.62**	**0.41**	**0.60**	**0.98**	**0.22**	**1.34**	**1.09**
1433	1432	*trans*-*α*-Bergamotene	0.00	0.00	0.00	0.02	0.01	0.00	0.01
1436	1436	*α*-Guaiene	0.00	0.02	0.00	0.00	0.09	0.00	0.00
1439	1442	6,9-Guaiadiene	0.00	0.00	0.00	0.00	0.00	0.00	0.00
1440	1439	(*Z*)-β-Farnesene	0.02	0.00	0.00	0.00	0.00	0.00	0.00
1444	1445	Selina-5,11-diene	0.00	0.00	0.00	0.00	0.00	0.00	0.00
1445	1447	*iso*-Germacrene D	0.02	0.03	0.02	0.00	0.01	0.00	0.00
1446	1446	*cis*-Muurola-3,5-diene	0.00	0.01	0.01	0.00	0.00	0.01	0.00
1450	1452	*trans*-Muurola-3,5-diene	0.01	0.02	0.02	0.04	0.01	0.06	0.04
1451	1452	(*E*)-β-Farnesene	0.00	0.00	0.00	0.00	0.00	0.00	0.00
1452	1453	ε-Muurolene	0.03	0.03	0.02	0.00	0.01	0.00	0.00
1452	1455	Sesquisabinene	0.01	0.00	0.00	0.00	0.00	0.00	0.00
1453	1455	Valerena-4,7(11)-diene	0.00	0.00	0.00	0.00	0.00	0.01	0.00
**1456**	**1454**	***α*-Humulene**	**0.35**	**0.53**	**0.27**	**0.54**	**0.10**	**0.20**	**0.38**
1459	1461	*cis*-Cadina-1(6),4-diene	0.00	0.00	0.00	0.00	0.00	0.00	0.00
1461	1464	9-*epi*-(*E*)-Caryophyllene	0.01	0.02	0.01	0.00	0.01	0.00	0.01
1463	1463	*cis*-Muurola-4(14),5-diene	0.02	0.05	0.03	0.01	0.01	0.01	0.01
1470	1471	4,5-di-*epi*-Aristolochene	0.00	0.00	0.00	0.00	0.00	0.00	0.00
1472	1472	*trans*-Cadina-1(6),4-diene	0.01	0.01	0.01	0.05	0.00	0.05	0.03
1473	1475	Selina-4,11-diene	0.00	0.00	0.00	0.00	0.00	0.00	0.00
**1475**	**1478**	**γ-Muurolene**	**0.20**	**0.16**	**0.21**	**0.33**	**0.07**	**0.49**	**0.35**
1478	1480	*cis*-4,10-Epoxyamorphane	0.08	0.03	0.04	0.16	0.02	0.27	0.16
1478	1483	*α*-Amorphene	0.00	0.00	0.00	0.00	0.00	0.00	0.00
**1482**	**1480**	**Germacrene D**	**6.46**	**7.75**	**5.07**	**0.04**	**1.76**	**0.12**	**0.13**
1487	1488	δ-Selinene	0.00	0.00	0.00	0.00	0.00	0.00	0.00
1488	1491	Eremophilene	0.00	0.00	0.00	0.00	0.00	0.00	0.00
1490	1487	β-Selinene	0.00	0.01	0.00	0.01	0.02	0.01	0.00
1493	1490	γ-Amorphene	0.04	0.08	0.06	0.06	0.02	0.10	0.06
1496	1497	*α*-Selinene	0.00	0.00	0.00	0.00	0.00	0.00	0.00
1496	1498	*epi*-Cubebol	0.00	0.00	0.00	0.09	0.00	0.19	0.09
1497	1498	Bicyclogermacrene	0.13	0.16	0.12	0.00	0.05	0.00	0.00

RIcalc	RIdb	Compound	LD190910G	LD190910H	LD190910I	LD190910J	LD190910K	LD190910L	LD190910M
**1499**	**1497**	**α-Muurolene**	**0.24**	**0.18**	**0.24**	**0.42**	**0.08**	**0.64**	**0.43**
1502	1497	Valencene	0.00	0.00	0.00	0.00	0.00	0.00	0.00
1502	1504	*iso*-Daucene	0.00	0.00	0.00	0.10	0.00	0.00	0.00
1503	1503	(*E*,*E*)-α-Farnesene	0.03	0.11	0.06	0.00	0.00	0.00	0.00
1503	1506	δ-Amorphene	0.00	0.00	0.00	0.00	0.00	0.01	0.00
1505	1505	α-Bulnesene	0.00	0.00	0.00	0.00	0.08	0.00	0.00
1508	1508	β-Bisabolene	0.07	0.00	0.00	0.01	0.00	0.00	0.00
1509	1511	Germacrene A	0.00	0.00	0.00	0.00	0.01	0.00	0.00
1514	1512	γ-Cadinene	0.03	0.05	0.03	0.00	0.01	0.01	0.01
**1516**	**1519**	**Cubebol**	**0.22**	**0.32**	**0.22**	**0.15**	**0.07**	**0.40**	**0.24**
**1518**	**1518**	**δ-Cadinene**	**0.27**	**0.43**	**0.36**	**0.31**	**0.14**	**0.48**	**0.31**
1521	1519	*trans*-Calamenene	0.00	0.00	0.00	0.00	0.00	0.02	0.00
1523	1521	Zonarene	0.00	0.02	0.01	0.04	0.00	0.06	0.03
1525	1524	β-Sesquiphellandrene	0.03	0.00	0.00	0.00	0.00	0.00	0.00
1533	1536	*trans*-Cadina-1,4-diene	0.02	0.03	0.02	0.04	0.01	0.06	0.03
1537	1538	α-Cadinene	0.01	0.03	0.02	0.00	0.01	0.00	0.00
1541	1541	α-Calacorene	0.00	0.00	0.00	0.00	0.00	0.00	0.00
1548	1546	α-Elemol	0.03	0.03	0.02	0.01	0.01	0.01	0.00
1559	1557	Germacrene B	0.00	0.01	0.01	0.01	0.01	0.02	0.01
1570	1566	1,5-Epoxysalvial-4(14)ene	0.01	0.02	0.02	0.00	0.01	0.01	0.00
1577	1576	Spathulenol	0.00	0.00	0.00	0.00	0.00	0.01	0.00
1578	1575	Germacra-1(10),5-dien-4β-ol	0.12	0.17	0.11	0.04	0.00	0.01	0.03
**1584**	**1587**	**Caryophyllene oxide**	**0.42**	**1.21**	**0.50**	**1.15**	**0.15**	**0.90**	**0.33**
1596	1596	Salvial-4(14)-en-1-one	0.00	0.03	0.02	0.00	0.01	0.01	0.00
1600	1600	Hexadecane	0.00	0.00	0.00	0.00	0.00	0.02	0.00
1615	1613	Humulene epoxide II	0.00	0.02	0.01	0.02	0.00	0.02	0.00
1628	1623	Humulane-1,6-dien-3-ol	0.07	0.12	0.07	0.00	0.01	0.03	0.03
1628	1627	Germacra-1(10),5-dien-4α-ol	0.00	0.00	0.00	0.00	0.00	0.05	0.02
1634	1631	1-*epi*-Cubenol	0.00	0.00	0.00	0.00	0.00	0.07	0.04
1634	---	Unidentified	0.00	0.00	0.00	0.00	0.00	0.00	0.17
1635	1629	*iso*-Spathulenol	0.00	0.00	0.00	0.00	0.00	0.00	0.00
1640	1633	γ-Eudesmol	0.00	0.00	0.00	0.00	0.00	0.00	0.00
1640	1642	Caryophylla-4(12),8(13)-dien-5α-ol	0.00	0.00	0.00	0.00	0.00	0.00	0.00
1641	1644	Caryophylla-4(12),8(13)-dien-5β-ol	0.00	0.00	0.00	0.03	0.00	0.00	0.00
1642	1638	(2*S*,5*E*)-Caryophyll-5-en-12-al	0.00	0.00	0.00	0.02	0.00	0.00	0.00
1647	1643	Cubenol	0.00	0.00	0.00	0.05	0.00	0.07	0.03
**1650**	**1651**	**α-Muurolol**	**0.51**	**0.43**	**0.51**	**1.06**	**0.15**	**1.93**	**1.18**
1658	1655	α-Eudesmol	0.00	0.00	0.00	0.00	0.00	0.00	0.00
1658	1655	α-Cadinol	0.00	0.00	0.00	0.00	0.00	0.00	0.00
1658	1656	β-Eudesmol	0.00	0.00	0.00	0.02	0.00	0.03	0.03
1660	1660	Selin-11-en-4α-ol	0.00	0.04	0.02	0.01	0.02	0.02	0.01
1671	1666	14-Hydroxy-9-*epi*-(*E*)-caryophyllene	0.00	0.00	0.00	0.05	0.00	0.07	0.00
1681	1676	Bornyl 8-hydroxyisobutanoate	0.00	0.00	0.00	0.00	0.00	0.00	0.00
2052	2050	Bornyl hydrocinnamate	0.00	0.00	0.00	0.02	0.00	0.09	0.03
2151	2152	3-Phenylpropyl 3-phenylpropanoate	0.00	0.00	0.00	0.04	0.00	0.24	0.02
**2224**	**2223**	**Bornyl cinnamate**	**0.47**	**0.71**	**0.84**	**0.56**	**0.76**	**1.20**	**0.12**
2318	2321	3-Phenylpropyl cinnamate	0.00	0.00	0.03	0.01	0.00	0.06	0.00
2434	2432	Phenylallyl cinnamate	0.00	0.04	0.03	0.01	0.00	0.00	0.00
		Monoterpene hydrocarbons	70.23	59.76	74.87	63.30	85.16	69.51	65.65
		Oxygenated monoterpenoids	2.76	3.36	2.00	3.92	5.84	9.80	8.46
		Sesquiterpene hydrocarbons	24.82	33.38	20.35	28.32	7.60	13.89	22.56
		Oxygenated sesquiterpenoids	1.46	2.43	1.54	2.85	0.46	4.11	2.19

RIcalc	RIdb	Compound	LD1909 10G	LD1909 10H	LD190 910I	LD190 910J	LD190 910K	LD190 910L	LD1909 10M
		Benzenoid aromatics	0.54	0.77	0.97	1.19	0.80	2.05	0.85
		Others	0.00	0.00	0.00	0.00	0.00	0.02	0.00
		Total identified	99.80	99.70	99.73	99.57	99.86	99.38	99.72

RI_{calc}: Retention indices determined with respect to a homologous series of *n*-alkanes on a ZB-5ms column. RI_{db}: Retention indices from the databases. Compounds in **boldface** were used in the multivariate (HCA and PCA) analyses.

Table S1. Continued.

RIcalc	RIdb	Compound	LD1909 10N	LD1909 10O	LD1909 10P	LD1909 10Q	LD1909 10R	LD1909 10S	LD1909 10T
763	763	Toluene	0.00	0.00	0.00	0.00	0.00	0.00	0.31
863	857	Ethylbenzene	0.00	0.00	0.03	0.07	0.00	0.00	0.09
893	891	Styrene	0.04	0.14	0.13	0.22	0.30	0.26	2.18
900	893	2-Bornene	0.00	0.00	0.00	0.00	0.00	0.00	0.72
908	910	(*E*)-2,6-Dimethyl-4-octene	0.00	0.00	0.00	0.00	0.00	0.00	0.00
920	919	5,5-Dimethyl-1-vinylbicyclo[2.1.1]hexane	0.00	0.00	0.00	0.00	0.00	0.00	0.00
923	922	Tricyclene	0.25	0.24	0.20	0.07	0.01	0.13	1.54
929	927	α-Thujene	0.32	0.32	0.29	0.08	0.02	0.23	0.12
**938**	**933**	**α-Pinene**	**20.61**	**20.91**	**19.20**	**5.75**	**0.56**	**11.76**	**9.46**
945	948	β-Citronellene	0.00	0.00	0.01	0.00	0.00	0.00	0.00
948	947	Allylbenzene	0.02	0.04	0.13	0.20	0.00	0.03	0.53
951	948	α-Fenchene	0.00	0.00	0.00	0.01	0.00	0.00	0.00
**953**	**953**	**Camphene**	**10.84**	**9.38**	**8.36**	**3.04**	**0.31**	**5.21**	**4.47**
956	953	Thuja-2,4(10)-diene	0.01	0.01	0.01	0.01	0.00	0.00	0.05
964	964	Benzaldehyde	0.00	0.00	0.00	0.00	0.00	0.00	0.02
966	964	(*Z*)-3,7-Dimethyl-2-octene	0.00	0.01	0.05	0.02	0.00	0.01	0.03
**973**	**972**	**Sabinene**	**2.00**	**1.58**	**1.36**	**0.35**	**0.08**	**1.08**	**0.64**
**979**	**978**	**β-Pinene**	**16.26**	**14.93**	**13.99**	**4.50**	**0.57**	**9.18**	**7.29**
982	988	α-Methylstyrene	0.00	0.00	0.00	0.00	0.00	0.00	0.02
**987**	**991**	**Myrcene**	**1.18**	**0.32**	**0.09**	**0.07**	**0.07**	**0.41**	**0.11**
989	990	2,3-Dehydro-1,8-cineole	0.02	0.01	0.01	0.01	0.02	0.01	0.01
991	990	*o*-Methylstyrene	0.00	0.00	0.00	0.00	0.00	0.00	0.09
999	997	*p*-Menth-2-ene	0.01	0.03	0.24	0.08	0.00	0.04	0.04
1002	1004	*p*-Menth-1(7),8-diene	0.00	0.00	0.00	0.00	0.00	0.00	0.00
1005	1007	α-Phellandrene	0.02	0.03	0.05	0.02	0.03	0.00	0.03
1007	1008	δ-3-Carene	0.01	0.01	0.01	0.00	0.00	0.01	0.00

RI_{calc}	RI_{db}	Compound	LD1909 10N	LD1909 10O	LD1909 10P	LD1909 10Q	LD1909 10R	LD1909 10S	LD1909 10T
1015	1018	α-Terpinene	0.05	0.05	0.18	0.06	0.00	0.07	0.06
**1021**	**1022**	**(2*E*,4*E*)-3,7-Dimethylocta-2,4-diene**	**0.42**	**0.51**	**0.99**	**0.16**	**0.48**	**0.00**	**0.57**
**1023**	**1025**	***p*-Cymene**	**0.11**	**0.26**	**1.86**	**0.73**	**0.00**	**0.28**	**0.53**
1026	1028	Indane	0.00	0.00	0.00	0.02	0.00	0.00	0.65
**1028**	**1030**	**Limonene**	**7.85**	**2.45**	**2.65**	**0.86**	**0.16**	**1.85**	**1.27**
1030	1031	β-Phellandrene	0.17	0.16	0.29	0.06	0.02	0.22	0.15
1031	1032	1,8-Cineole	0.14	0.08	0.12	0.03	0.00	0.05	0.02
1034	1034	(*Z*)-β-Ocimene	0.02	0.01	0.02	0.01	0.00	0.01	0.01
1044	1046	(*E*)-β-Ocimene	0.03	0.05	0.05	0.01	0.03	0.00	0.07
1057	1058	γ-Terpinene	0.08	0.10	0.32	0.10	0.01	0.10	0.13
1060	1064	Benzenemethanol	0.00	0.00	0.00	0.01	0.00	0.00	0.00
1066	1068	Acetophenone	0.00	0.20	0.23	0.33	0.19	0.14	0.11
1069	1069	*cis*-Sabinene hydrate	0.05	0.03	0.03	0.02	0.00	0.02	0.03
1085	1086	Terpinolene	0.16	0.08	0.13	0.04	0.01	0.11	0.06
1089	1090	Fenchone	0.00	0.00	0.01	0.00	0.00	0.00	0.00
1091	1093	*p*-Cymenene	0.00	0.00	0.00	0.00	0.00	0.00	0.02
1099	1098	Perillene	0.00	0.00	0.00	0.00	0.00	0.00	0.00
1099	1101	α-Pinene oxide	0.00	0.00	0.00	0.00	0.00	0.00	0.00
1100	1101	Linalool	0.08	0.02	0.02	0.02	0.01	0.04	0.03
1101	1101	*trans*-Sabinene hydrate	0.02	0.01	0.01	0.00	0.00	0.01	0.00
1121	1121	*exo*-Fenchol (= β-Fenchol)	0.00	0.00	0.00	0.00	0.00	0.00	0.00
1123	1122	Chrystanthenone	0.00	0.00	0.00	0.00	0.00	0.00	0.00
1123	1122	*trans*-*p*-Mentha-2,8-dien-1-ol	0.00	0.00	0.00	0.00	0.00	0.00	0.00
1125	1224	*p*-Menth-1-en-9-al	0.00	0.00	0.00	0.00	0.00	0.00	0.06
1126	1124	*cis*-*p*-Menth-2-en-1-ol	0.01	0.00	0.01	0.00	0.00	0.00	0.00
1128	1127	α-Campholenal	0.00	0.00	0.00	0.00	0.00	0.00	0.00
1134	1134	*cis*-Limonene oxide	0.00	0.00	0.00	0.00	0.00	0.00	0.00
1143	1141	*trans*-Pinocarveol	0.01	0.00	0.00	0.00	0.00	0.00	0.00
1144	1141	*cis*-Verbenol	0.00	0.00	0.00	0.00	0.00	0.00	0.00
1145	1142	*trans*-*p*-Menth-2-en-1-ol	0.01	0.00	0.01	0.00	0.00	0.00	0.00
1145	1143	*trans*-Dihydro-β-terpineol	0.00	0.00	0.00	0.00	0.00	0.00	0.00
1148	1146	*trans*-Verbenol	0.03	0.02	0.00	0.00	0.00	0.00	0.00

RI_{calc}	RI_{db}	Compound	LD1909 10N	LD1909 10O	LD1909 10P	LD1909 10Q	LD1909 10R	LD1909 10S	LD1909 10T
**1150**	**1149**	**Camphor**	**2.10**	**1.78**	**2.38**	**2.56**	**0.10**	**1.36**	**0.96**
1151	1149	*trans*-Dihydro-α-terpineol	0.00	0.00	0.00	0.00	0.00	0.00	0.00
1152	1150	α-Phellandren-8-ol	0.00	0.00	0.00	0.00	0.00	0.00	0.00
1158	1156	Camphene hydrate	0.00	0.01	0.01	0.01	0.00	0.00	0.00
1159	1157	Sabina ketone	0.00	0.00	0.00	0.00	0.00	0.00	0.00
1160	1158	Menthone	0.00	0.00	0.00	0.00	0.00	0.00	0.00
1163	1160	*trans*-Pinocamphone	0.00	0.01	0.00	0.00	0.00	0.00	0.00
1165	1164	Pinocarvone	0.01	0.01	0.01	0.00	0.00	0.00	0.00
1167	1164	*cis*-Dihydro-α-terpineol	0.00	0.00	0.00	0.00	0.00	0.00	0.00
1167	1165	Isoborneol	0.02	0.01	0.01	0.01	0.00	0.01	0.00
1170	1171	4-Ethylphenol	0.00	0.00	0.00	0.00	0.01	0.00	0.00
**1175**	**1173**	**Borneol**	**0.80**	**0.57**	**0.57**	**0.84**	**0.11**	**0.63**	**0.40**
1179	1176	*cis*-Pinocamphone	0.01	0.02	0.01	0.01	0.00	0.00	0.00
1181	1179	*iso*-Menthol	0.00	0.00	0.00	0.00	0.00	0.00	0.00
1181	1179	2-Isopropenyl-5-methyl-4-hexenal	0.00	0.00	0.00	0.00	0.00	0.00	0.00
1184	1180	Terpinen-4-ol	0.21	0.14	0.24	0.14	0.02	0.13	0.12
1187	1183	Myrtanal	0.00	0.00	0.00	0.00	0.00	0.00	0.00
1190	1187	Cryptone	0.00	0.00	0.00	0.00	0.00	0.00	0.00
1190	1189	*p*-Cymen-8-ol	0.01	0.00	0.00	0.00	0.00	0.00	0.00
1198	1202	Myrtenol	0.00	0.00	0.00	0.00	0.00	0.00	0.00
1199	1195	α-Terpineol	0.54	0.22	0.35	0.26	0.05	0.26	0.23
1202	1198	(*Z*)-Dihydrocarvone	0.00	0.00	0.00	0.00	0.00	0.00	0.00
1202	1201	Estragole (= Methyl chavicol)	0.00	0.00	0.00	0.00	0.00	0.00	0.00
1211	1208	Verbenone	0.02	0.02	0.01	0.01	0.00	0.00	0.00
1234	1235	Hydrocinnamic alcohol (= Benzenepropanol)	0.08	0.04	0.00	0.17	0.33	0.03	0.10
1235	1232	*cis*-Carveol	0.02	0.00	0.00	0.00	0.00	0.00	0.00
1247	1246	Carvone	0.00	0.00	0.00	0.00	0.00	0.00	0.00
1250	1246	*trans*-Shisool	0.00	0.00	0.00	0.00	0.00	0.00	0.00
1274	1276	2,3-Pinanediol	0.00	0.00	0.00	0.00	0.00	0.00	0.00
1278	1273	(*E*)-Cinnamaldehyde	0.00	0.01	0.02	0.06	0.02	0.02	0.00
1280	1278	Perilla aldehyde	0.00	0.03	0.00	0.00	0.00	0.00	0.00
1283	1278	*cis*-Verbenyl acetate	0.00	0.00	0.00	0.00	0.00	0.00	0.00

RI_{calc}	RI_{db}	Compound	LD1909 10N	LD1909 10O	LD1909 10P	LD1909 10Q	LD1909 10R	LD1909 10S	LD1909 10T
**1286**	**1285**	**Bornyl acetate**	**8.69**	**0.23**	**0.24**	**1.26**	**0.24**	**2.98**	**0.38**
1287	1287	Isobornyl acetate	0.00	0.00	0.00	0.00	0.00	0.00	0.00
1300	1297	*p*-Menth-1-en-9-ol	0.00	0.00	0.00	0.02	0.00	0.00	0.00
1300	1299	Perilla alcohol	0.00	0.01	0.01	0.04	0.00	0.00	0.00
1300	1300	Carvacrol	0.00	0.00	0.00	0.00	0.00	0.00	0.00
1309	1309	(*E*)-Cinnamyl alcohol	0.00	0.07	0.00	0.15	0.05	0.04	0.00
1331	1334	Bicycloelemene	0.00	0.00	0.00	0.04	0.00	0.00	0.03
1334	1335	δ-Elemene	0.00	0.02	0.00	0.01	0.01	0.00	0.00
**1349**	**1349**	***α*-Cubebene**	**0.24**	**0.54**	**0.30**	**1.12**	**0.43**	**0.48**	**0.42**
1352	1352	Ethyl hydrocinnamate	0.00	0.00	0.00	0.00	0.01	0.00	0.00
1371	1367	Cyclosativene	0.18	0.22	0.18	0.42	0.26	0.31	0.23
1377	1375	*α*-Copaene	0.09	0.17	0.30	0.62	0.28	0.21	0.15
1380	1384	Daucene	0.00	0.00	0.00	0.01	0.00	0.00	0.02
1383	1383	*cis*-β-Elemene	0.00	0.07	0.00	0.01	0.02	0.01	0.01
1383	1385	Isopropyl 3-phenylpropanoate	0.00	0.00	0.00	0.00	0.00	0.00	0.00
1385	1382	β-Bourbonene	0.00	0.04	0.04	0.08	0.02	0.02	0.01
**1389**	**1392**	**β-Cubebene**	**1.18**	**1.57**	**1.59**	**3.01**	**3.43**	**2.82**	**2.59**
**1391**	**1390**	***trans*-β-Elemene**	**0.05**	**1.47**	**0.08**	**0.17**	**0.33**	**0.15**	**0.14**
1394	1394	Sativene	0.00	0.00	0.00	0.02	0.01	0.01	0.01
1406	1405	(*Z*)-Caryophyllene	0.01	0.02	0.01	0.02	0.02	0.01	0.02
**1423**	**1424**	**(*E*)-Caryophyllene**	**17.19**	**26.14**	**28.43**	**41.99**	**64.38**	**42.64**	**45.53**
1423	1428	β-Duprezianene	0.00	0.00	0.00	0.00	0.00	0.00	0.00
1430	1432	γ-Elemene	0.00	0.00	0.00	0.00	0.08	0.00	0.00
**1431**	**1433**	**β-Copaene**	**1.54**	**2.30**	**1.92**	**4.88**	**4.16**	**3.36**	**2.72**
1433	1432	*trans*-*α*-Bergamotene	0.01	0.00	0.02	0.00	0.00	0.00	0.00
1436	1436	*α*-Guaiene	0.00	0.37	0.00	0.00	0.00	0.00	0.00
1439	1442	6,9-Guaiadiene	0.00	0.00	0.00	0.00	0.00	0.00	0.00
1440	1439	(*Z*)-β-Farnesene	0.01	0.00	0.00	0.00	0.00	0.00	0.00
1444	1445	Selina-5,11-diene	0.00	0.00	0.00	0.00	0.00	0.00	0.00
1445	1447	*iso*-Germacrene D	0.00	0.00	0.00	0.03	0.00	0.00	0.00
1446	1446	*cis*-Muurola-3,5-diene	0.00	0.00	0.00	0.04	0.03	0.01	0.01
1450	1452	*trans*-Muurola-3,5-diene	0.04	0.08	0.07	0.28	0.26	0.09	0.09

RI_{calc}	RI_{db}	Compound	LD1909 10N	LD1909 10O	LD1909 10P	LD1909 10Q	LD1909 10R	LD1909 10S	LD1909 10T
1451	1452	(*E*)-β-Farnesene	0.00	0.00	0.00	0.00	0.00	0.00	0.00
1452	1453	ε-Muurolene	0.00	0.00	0.00	0.00	0.00	0.00	0.00
1452	1455	Sesquisabinene	0.01	0.00	0.03	0.00	0.03	0.01	0.01
1453	1455	Valerena-4,7(11)-diene	0.00	0.00	0.00	0.00	0.00	0.00	0.00
**1456**	**1454**	**α-Humulene**	**0.38**	**0.70**	**0.66**	**1.11**	**2.03**	**1.14**	**1.20**
1459	1461	*cis*-Cadina-1(6),4-diene	0.00	0.00	0.00	0.04	0.00	0.00	0.00
1461	1464	9-*epi*-(*E*)-Caryophyllene	0.01	0.00	0.01	0.00	0.01	0.01	0.01
1463	1463	*cis*-Muurola-4(14),5-diene	0.01	0.02	0.04	0.14	0.11	0.03	0.03
1470	1471	4,5-di-*epi*-Aristolochene	0.00	0.00	0.00	0.00	0.00	0.00	0.00
1472	1472	*trans*-Cadina-1(6),4-diene	0.03	0.06	0.07	0.23	0.14	0.09	0.06
1473	1475	Selina-4,11-diene	0.00	0.00	0.00	0.00	0.00	0.00	0.00
**1475**	**1478**	**γ-Muurolene**	**0.49**	**0.81**	**0.67**	**1.87**	**1.76**	**1.14**	**1.02**
1478	1480	*cis*-4,10-Epoxyamorphane	0.26	0.36	0.28	0.61	0.58	0.28	0.41
1478	1483	α-Amorphene	0.00	0.00	0.00	0.00	0.00	0.00	0.00
**1482**	**1480**	**Germacrene D**	**0.26**	**0.64**	**3.23**	**5.49**	**4.41**	**0.98**	**0.68**
1487	1488	δ-Selinene	0.00	0.00	0.00	0.00	0.00	0.00	0.00
1488	1491	Eremophilene	0.00	0.00	0.00	0.00	0.00	0.00	0.00
1490	1487	β-Selinene	0.00	0.16	0.01	0.03	0.02	0.01	0.01
1493	1490	γ-Amorphene	0.08	0.16	0.15	0.61	0.52	0.20	0.19
1496	1497	α-Selinene	0.00	0.00	0.00	0.00	0.00	0.00	0.00
1496	1498	*epi*-Cubebol	0.11	0.33	0.19	0.58	0.47	0.26	0.23
1497	1498	Bicyclogermacrene	0.00	0.00	0.00	0.00	0.06	0.00	0.00
**1499**	**1497**	**α-Muurolene**	**0.64**	**1.02**	**0.83**	**2.39**	**2.44**	**1.49**	**1.38**
1502	1497	Valencene	0.00	0.00	0.00	0.13	0.00	0.05	0.00
1502	1504	*iso*-Daucene	0.00	0.00	0.09	0.00	0.00	0.00	0.00
1503	1503	(*E*,*E*)-α-Farnesene	0.04	0.00	0.05	0.00	0.10	0.00	0.11
1503	1506	δ-Amorphene	0.00	0.00	0.00	0.00	0.00	0.00	0.00
1505	1505	α-Bulnesene	0.00	0.31	0.01	0.03	0.00	0.02	0.00
1508	1508	β-Bisabolene	0.05	0.00	0.00	0.00	0.00	0.00	0.00
1509	1511	Germacrene A	0.00	0.00	0.00	0.00	0.00	0.00	0.00
1514	1512	γ-Cadinene	0.00	0.00	0.04	0.10	0.07	0.03	0.02
**1516**	**1519**	**Cubebol**	**0.31**	**0.51**	**0.39**	**1.23**	**1.38**	**0.68**	**0.78**

RI$_{calc}$	RI$_{db}$	Compound	LD1909 10N	LD1909 10O	LD1909 10P	LD1909 10Q	LD1909 10R	LD1909 10S	LD1909 10T
**1518**	**1518**	**δ-Cadinene**	**0.35**	**0.64**	**0.64**	**2.15**	**1.59**	**0.88**	**0.74**
1521	1519	*trans*-Calamenene	0.00	0.00	0.00	0.13	0.00	0.00	0.00
1523	1521	Zonarene	0.04	0.06	0.05	0.21	0.14	0.09	0.06
1525	1524	β-Sesquiphellandrene	0.00	0.00	0.00	0.00	0.00	0.00	0.00
1533	1536	*trans*-Cadina-1,4-diene	0.04	0.08	0.07	0.25	0.23	0.09	0.09
1537	1538	α-Cadinene	0.00	0.00	0.02	0.04	0.04	0.01	0.00
1541	1541	α-Calacorene	0.00	0.00	0.00	0.00	0.00	0.00	0.00
1548	1546	α-Elemol	0.02	0.01	0.01	0.02	0.14	0.02	0.02
1559	1557	Germacrene B	0.02	0.06	0.03	0.08	0.11	0.05	0.05
1570	1566	1,5-Epoxysalvial-4(14)ene	0.00	0.00	0.00	0.00	0.00	0.00	0.00
1577	1576	Spathulenol	0.00	0.00	0.03	0.00	0.00	0.00	0.00
1578	1575	Germacra-1(10),5-dien-4β-ol	0.00	0.00	0.00	0.06	0.06	0.06	0.03
**1584**	**1587**	**Caryophyllene oxide**	**0.75**	**1.23**	**0.51**	**0.74**	**0.80**	**0.98**	**1.02**
1596	1596	Salvial-4(14)-en-1-one	0.00	0.00	0.01	0.02	0.01	0.00	0.00
1600	1600	Hexadecane	0.00	0.00	0.00	0.00	0.00	0.00	0.00
1615	1613	Humulene epoxide II	0.02	0.03	0.01	0.02	0.02	0.02	0.03
1628	1623	Humulane-1,6-dien-3-ol	0.00	0.02	0.37	0.62	0.10	0.03	0.03
1628	1627	Germacra-1(10),5-dien-4α-ol	0.03	0.04	0.03	0.09	0.12	0.07	0.12
1634	1631	1-*epi*-Cubenol	0.00	0.00	0.00	0.18	0.00	0.09	0.07
1634	---	Unidentified	0.29	0.59	0.45	0.86	1.29	0.56	0.77
1635	1629	*iso*-Spathulenol	0.00	0.00	0.00	0.00	0.00	0.00	0.00
1640	1633	γ-Eudesmol	0.00	0.00	0.00	0.00	0.00	0.00	0.02
1640	1642	Caryophylla-4(12),8(13)-dien-5α-ol	0.00	0.00	0.00	0.00	0.00	0.00	0.00
1641	1644	Caryophylla-4(12),8(13)-dien-5β-ol	0.00	0.00	0.00	0.00	0.00	0.00	0.00
1642	1638	(2*S*,5*E*)-Caryophyll-5-en-12-al	0.00	0.00	0.00	0.00	0.00	0.00	0.00
1647	1643	Cubenol	0.04	0.08	0.07	0.21	0.18	0.12	0.11
**1650**	**1651**	**α-Muurolol**	**1.34**	**2.51**	**1.97**	**3.79**	**3.36**	**2.45**	**3.36**
1658	1655	α-Eudesmol	0.00	0.00	0.00	0.00	0.00	0.00	0.00
1658	1655	α-Cadinol	0.00	0.00	0.00	0.00	0.00	0.06	0.00
1658	1656	β-Eudesmol	0.00	0.00	0.03	0.09	0.07	0.00	0.05
1660	1660	Selin-11-en-4α-ol	0.00	0.57	0.02	0.03	0.02	0.02	0.03
1671	1666	14-Hydroxy-9-*epi*-(*E*)-caryophyllene	0.00	0.00	0.00	0.00	0.00	0.00	0.00

RI_{calc}	RI_{db}	Compound	LD1909 10N	LD1909 10O	LD1909 10P	LD1909 10Q	LD1909 10R	LD1909 10S	LD1909 10T
1681	1676	Bornyl 8-hydroxyisobutanoate	0.00	0.00	0.00	0.00	0.00	0.00	0.00
2052	2050	Bornyl hydrocinnamate	0.02	0.01	0.02	0.02	0.00	0.00	0.02
2151	2152	3-Phenylpropyl 3-phenylpropanoate	0.02	0.02	0.00	0.03	0.02	0.00	0.04
**2224**	**2223**	**Bornyl cinnamate**	**0.43**	**0.60**	**0.81**	**0.23**	**0.25**	**0.16**	**0.95**
2318	2321	3-Phenylpropyl cinnamate	0.03	0.02	0.00	0.03	0.00	0.00	0.10
2434	2432	Phenylallyl cinnamate	0.00	0.00	0.00	0.00	0.00	0.00	0.04
		Monoterpene hydrocarbons	60.39	51.45	50.34	16.03	2.36	30.68	27.35
		Oxygenated monoterpenoids	12.81	3.23	4.07	5.24	0.55	5.51	2.22
		Sesquiterpene hydrocarbons	22.98	37.73	39.64	67.79	87.54	56.43	57.60
		Oxygenated sesquiterpenoids	2.87	5.70	3.93	8.28	7.30	5.15	6.26
		Benzenoid aromatics	0.63	1.16	1.36	1.54	1.17	0.70	5.23
		Others	0.00	0.00	0.00	0.00	0.00	0.00	0.00
		Total identified	99.67	99.27	99.34	98.88	98.93	98.46	98.64

RI_{calc}: Retention indices determined with respect to a homologous series of *n*-alkanes on a ZB-5ms column. RI_{db}: Retention indices from the databases. Compounds in **boldface** were used in the multivariate (HCA and PCA) analyses.

Table S1. Continued.

RI_{calc}	RI_{db}	Compound	LD190910U	LD190910V	LD190910W	LD190910X
763	763	Toluene	0.00	0.00	0.00	0.00
863	857	Ethylbenzene	0.00	0.00	0.00	0.00
893	891	Styrene	0.06	0.11	0.06	0.00
900	893	2-Bornene	0.00	0.00	0.00	0.00
908	910	(*E*)-2,6-Dimethyl-4-octene	0.00	0.00	0.00	0.00
920	919	5,5-Dimethyl-1-vinylbicyclo[2.1.1]hexane	0.00	0.00	0.00	0.00
923	922	Tricyclene	0.23	0.18	0.26	0.01
929	927	α-Thujene	0.28	0.27	0.54	0.05
**938**	**933**	**α-Pinene**	**15.27**	**15.67**	**29.07**	**3.82**
945	948	β-Citronellene	0.00	0.05	0.00	0.00
948	947	Allylbenzene	0.05	0.51	0.05	0.02
951	948	α-Fenchene	0.01	0.01	0.00	0.01
**953**	**953**	**Camphene**	**8.54**	**7.71**	**10.99**	**0.34**
956	953	Thuja-2,4(10)-diene	0.00	0.01	0.00	0.00
964	964	Benzaldehyde	0.00	0.00	0.00	0.00
966	964	(*Z*)-3,7-Dimethyl-2-octene	0.02	0.12	0.07	0.08
**973**	**972**	**Sabinene**	**1.46**	**0.43**	**2.35**	**0.02**
**979**	**978**	**β-Pinene**	**10.92**	**11.71**	**26.03**	**5.65**
982	988	α-Methylstyrene	0.00	0.00	0.00	0.00
**987**	**991**	**Myrcene**	**0.27**	**0.33**	**5.12**	**1.34**
989	990	2,3-Dehydro-1,8-cineole	0.00	0.00	0.00	0.00
991	990	*o*-Methylstyrene	0.00	0.00	0.00	0.00
999	997	*p*-Menth-2-ene	0.04	0.10	0.02	0.13
1002	1004	*p*-Menth-1(7),8-diene	0.00	0.00	0.01	0.00
1005	1007	α-Phellandrene	0.03	0.05	0.03	0.01
1007	1008	δ-3-Carene	0.01	0.01	0.01	0.00
1015	1018	α-Terpinene	0.06	0.14	0.06	0.02
**1021**	**1022**	**(2*E*,4*E*)-3,7-Dimethylocta-2,4-diene**	**0.89**	**0.19**	**0.08**	**0.36**
**1023**	**1025**	***p*-Cymene**	**0.41**	**1.29**	**0.28**	**0.58**
1026	1028	Indane	0.00	0.02	0.00	0.00
**1028**	**1030**	**Limonene**	**5.65**	**2.97**	**6.75**	**0.57**
1030	1031	β-Phellandrene	0.18	0.11	0.12	0.08
1031	1032	1,8-Cineole	0.15	0.09	0.15	0.03
1034	1034	(*Z*)-β-Ocimene	0.02	0.03	0.02	0.02
1044	1046	(*E*)-β-Ocimene	0.07	0.07	0.04	0.07
1057	1058	γ-Terpinene	0.13	0.30	0.13	0.10
1060	1064	Benzenemethanol	0.00	0.00	0.00	0.00

RI_{calc}	RI_{db}	Compound	LD190910U	LD190910V	LD190910W	LD190910X
1066	1068	Acetophenone	0.10	0.01	0.00	0.00
1069	1069	*cis*-Sabinene hydrate	0.03	0.03	0.04	0.00
1085	1086	Terpinolene	0.13	0.18	0.12	0.07
1089	1090	Fenchone	0.00	0.00	0.00	0.00
1091	1093	*p*-Cymenene	0.00	0.00	0.00	0.00
1099	1098	Perillene	0.00	0.00	0.02	0.00
1099	1101	α-Pinene oxide	0.00	0.00	0.00	0.00
1100	1101	Linalool	0.05	0.03	0.06	0.00
1101	1101	*trans*-Sabinene hydrate	0.01	0.02	0.03	0.00
1121	1121	*exo*-Fenchol (= β-Fenchol)	0.00	0.01	0.00	0.01
1123	1122	Chrystanthenone	0.00	0.00	0.00	0.00
1123	1122	*trans-p*-Mentha-2,8-dien-1-ol	0.00	0.00	0.00	0.00
1125	1224	*p*-Menth-1-en-9-al	0.00	0.01	0.00	0.00
1126	1124	*cis-p*-Menth-2-en-1-ol	0.00	0.01	0.00	0.00
1128	1127	α-Campholenal	0.00	0.00	0.01	0.00
1134	1134	*cis*-Limonene oxide	0.00	0.00	0.00	0.00
1143	1141	*trans*-Pinocarveol	0.00	0.02	0.02	0.00
1144	1141	*cis*-Verbenol	0.00	0.00	0.00	0.00
1145	1142	*trans-p*-Menth-2-en-1-ol	0.00	0.01	0.01	0.00
1145	1143	*trans*-Dihydro-β-terpineol	0.00	0.00	0.00	0.00
1148	1146	*trans*-Verbenol	0.00	0.02	0.02	0.00
**1150**	**1149**	**Camphor**	**1.02**	**0.23**	**0.04**	**0.04**
1151	1149	*trans*-Dihydro-α-terpineol	0.00	0.00	0.00	0.00
1152	1150	α-Phellandren-8-ol	0.00	0.00	0.00	0.00
1158	1156	Camphene hydrate	0.00	0.02	0.00	0.00
1159	1157	Sabina ketone	0.00	0.00	0.00	0.00
1160	1158	Menthone	0.00	0.00	0.00	0.00
1163	1160	*trans*-Pinocamphone	0.00	0.00	0.00	0.00
1165	1164	Pinocarvone	0.01	0.00	0.00	0.00
1167	1164	*cis*-Dihydro-α-terpineol	0.00	0.00	0.00	0.00
1167	1165	Isoborneol	0.00	0.02	0.00	0.00
1170	1171	4-Ethylphenol	0.00	0.00	0.00	0.00
**1175**	**1173**	**Borneol**	**0.37**	**0.28**	**0.15**	**0.02**
1179	1176	*cis*-Pinocamphone	0.00	0.00	0.00	0.00
1181	1179	*iso*-Menthol	0.00	0.00	0.00	0.00
1181	1179	2-Isopropenyl-5-methyl-4-hexenal	0.00	0.00	0.00	0.00
1184	1180	Terpinen-4-ol	0.17	0.31	0.17	0.14
1187	1183	Myrtanal	0.00	0.00	0.00	0.00
1190	1187	Cryptone	0.00	0.00	0.00	0.00
1190	1189	*p*-Cymen-8-ol	0.00	0.00	0.00	0.00
1198	1202	Myrtenol	0.00	0.00	0.00	0.00
1199	1195	α-Terpineol	0.38	0.33	0.27	0.16
1202	1198	(*Z*)-Dihydrocarvone	0.00	0.00	0.00	0.00
1202	1201	Estragole (= Methyl chavicol)	0.00	0.03	0.09	0.00
1211	1208	Verbenone	0.01	0.01	0.00	0.00
1234	1235	Hydrocinnamic alcohol (= Benzenepropanol)	0.05	0.03	0.00	0.00
1235	1232	*cis*-Carveol	0.00	0.00	0.00	0.00
1247	1246	Carvone	0.00	0.00	0.00	0.00
1250	1246	*trans*-Shisool	0.00	0.00	0.00	0.00
1274	1276	2,3-Pinanediol	0.00	0.00	0.00	0.00
1278	1273	(*E*)-Cinnamaldehyde	0.00	0.00	0.00	0.00
1280	1278	Perilla aldehyde	0.00	0.00	0.00	0.00
1283	1278	*cis*-Verbenyl acetate	0.00	0.00	0.00	0.01
**1286**	**1285**	**Bornyl acetate**	**0.76**	**6.12**	**6.28**	**1.58**
1287	1287	Isobornyl acetate	0.00	0.02	0.02	0.00
1300	1297	*p*-Menth-1-en-9-ol	0.00	0.00	0.00	0.00
1300	1299	Perilla alcohol	0.00	0.00	0.00	0.00
1300	1300	Carvacrol	0.00	0.00	0.00	0.00
1309	1309	(*E*)-Cinnamyl alcohol	0.00	0.00	0.00	0.00
1331	1334	Bicycloelemene	0.02	0.01	0.00	0.00
1334	1335	δ-Elemene	0.04	0.15	0.03	0.00
**1349**	**1349**	**α-Cubebene**	**0.25**	**0.54**	**0.10**	**1.41**
1352	1352	Ethyl hydrocinnamate	0.00	0.00	0.00	0.00
1371	1367	Cyclosativene	0.18	0.19	0.06	0.40
1377	1375	α-Copaene	0.85	0.11	0.11	0.50

RI$_{calc}$	RI$_{db}$	Compound	LD190910U	LD190910V	LD190910W	LD190910X
1380	1384	Daucene	0.01	0.01	0.00	0.00
1383	1383	*cis*-β-Elemene	0.17	0.00	0.00	0.00
1383	1385	Isopropyl 3-phenylpropanoate	0.00	0.00	0.00	0.00
1385	1382	β-Bourbonene	0.11	0.01	0.00	0.02
**1389**	**1392**	**β-Cubebene**	**1.84**	**0.73**	**0.37**	**0.11**
**1391**	**1390**	***trans*-β-Elemene**	**3.14**	**0.08**	**0.02**	**0.03**
1394	1394	Sativene	0.01	0.01	0.00	0.01
1406	1405	(*Z*)-Caryophyllene	0.01	0.05	0.00	0.05
**1423**	**1424**	**(*E*)-Caryophyllene**	**22.84**	**34.84**	**6.46**	**50.30**
1423	1428	β-Duprezianene	0.00	0.00	0.01	0.00
1430	1432	γ-Elemene	0.00	0.00	0.00	0.00
**1431**	**1433**	**β-Copaene**	**1.68**	**1.98**	**0.56**	**3.58**
1433	1432	*trans*-α-Bergamotene	0.03	0.00	0.02	0.00
1436	1436	α-Guaiene	0.42	0.00	0.00	0.00
1439	1442	6,9-Guaiadiene	0.00	0.01	0.00	0.00
1440	1439	(*Z*)-β-Farnesene	0.02	0.00	0.00	0.00
1444	1445	Selina-5,11-diene	0.00	0.00	0.00	0.00
1445	1447	*iso*-Germacrene D	0.04	0.00	0.00	0.00
1446	1446	*cis*-Muurola-3,5-diene	0.01	0.01	0.00	0.00
1450	1452	*trans*-Muurola-3,5-diene	0.07	0.08	0.01	0.16
1451	1452	(*E*)-β-Farnesene	0.00	0.00	0.00	0.00
1452	1453	ε-Muurolene	0.05	0.00	0.00	0.00
1452	1455	Sesquisabinene	0.02	0.00	0.00	0.00
1453	1455	Valerena-4,7(11)-diene	0.00	0.00	0.00	0.00
**1456**	**1454**	**α-Humulene**	**0.71**	**0.89**	**0.13**	**1.31**
1459	1461	*cis*-Cadina-1(6),4-diene	0.00	0.00	0.00	0.02
1461	1464	9-*epi*-(*E*)-Caryophyllene	0.02	0.00	0.01	0.00
1463	1463	*cis*-Muurola-4(14),5-diene	0.10	0.02	0.00	0.03
1470	1471	4,5-di-*epi*-Aristolochene	0.00	0.00	0.00	0.00
1472	1472	*trans*-Cadina-1(6),4-diene	0.04	0.06	0.01	0.24
1473	1475	Selina-4,11-diene	0.00	0.00	0.00	0.00
**1475**	**1478**	**γ-Muurolene**	**0.59**	**0.67**	**0.17**	**1.59**
1478	1480	*cis*-4,10-Epoxyamorphane	0.23	0.35	0.06	0.61
1478	1483	α-Amorphene	0.00	0.00	0.00	0.00
**1482**	**1480**	**Germacrene D**	**9.92**	**0.16**	**0.22**	**0.16**
1487	1488	δ-Selinene	0.00	0.02	0.00	0.02
1488	1491	Eremophilene	0.00	0.00	0.00	0.00
1490	1487	β-Selinene	0.13	0.03	0.00	0.14
1493	1490	γ-Amorphene	0.22	0.14	0.01	0.34
1496	1497	α-Selinene	0.00	0.00	0.00	0.00
1496	1498	*epi*-Cubebol	0.44	0.47	0.08	1.48
1497	1498	Bicyclogermacrene	0.00	0.00	0.00	0.00
**1499**	**1497**	**α-Muurolene**	**0.71**	**0.85**	**0.22**	**1.88**
1502	1497	Valencene	0.00	0.00	0.00	0.00
1502	1504	*iso*-Daucene	0.00	0.00	0.02	0.00
1503	1503	(*E*,*E*)-α-Farnesene	0.00	0.00	0.00	0.00
1503	1506	δ-Amorphene	0.00	0.03	0.00	0.05
1505	1505	α-Bulnesene	0.40	0.01	0.00	0.00
1508	1508	β-Bisabolene	0.11	0.00	0.00	0.00
1509	1511	Germacrene A	0.00	0.00	0.00	0.00
1514	1512	γ-Cadinene	0.08	0.01	0.00	0.17
**1516**	**1519**	**Cubebol**	**0.68**	**1.25**	**0.18**	**2.09**
**1518**	**1518**	**δ-Cadinene**	**0.87**	**0.60**	**0.13**	**2.24**
1521	1519	*trans*-Calamenene	0.00	0.00	0.00	0.39
1523	1521	Zonarene	0.07	0.09	0.00	0.15
1525	1524	β-Sesquiphellandrene	0.00	0.00	0.00	0.00
1533	1536	*trans*-Cadina-1,4-diene	0.08	0.08	0.01	0.26
1537	1538	α-Cadinene	0.04	0.00	0.00	0.00
1541	1541	α-Calacorene	0.00	0.00	0.00	0.07
1548	1546	α-Elemol	0.04	0.03	0.02	0.00
1559	1557	Germacrene B	0.09	0.03	0.00	0.00
1570	1566	1,5-Epoxysalvial-4(14)ene	0.02	0.00	0.00	0.00
1577	1576	Spathulenol	0.00	0.00	0.01	0.00
1578	1575	Germacra-1(10),5-dien-4β-ol	0.14	0.00	0.00	0.00
**1584**	**1587**	**Caryophyllene oxide**	**0.82**	**1.47**	**0.25**	**4.40**

RI_{calc}	RI_{db}	Compound	LD190910U	LD190910V	LD190910W	LD190910X
1596	1596	Salvial-4(14)-en-1-one	0.03	0.00	0.00	0.00
1600	1600	Hexadecane	0.00	0.00	0.00	0.00
1615	1613	Humulene epoxide II	0.03	0.04	0.00	0.00
1628	1623	Humulane-1,6-dien-3-ol	0.58	0.00	0.00	0.00
1628	1627	Germacra-1(10),5-dien-4α-ol	0.04	0.11	0.01	0.14
1634	1631	1-*epi*-Cubenol	0.06	0.00	0.00	0.33
1634	---	Unidentified	0.32	0.41	0.09	1.14
1635	1629	*iso*-Spathulenol	0.00	0.15	0.03	0.00
1640	1633	γ-Eudesmol	0.00	0.01	0.00	0.00
1640	1642	Caryophylla-4(12),8(13)-dien-5α-ol	0.00	0.00	0.00	0.00
1641	1644	Caryophylla-4(12),8(13)-dien-5β-ol	0.00	0.00	0.00	0.00
1642	1638	(2*S*,5*E*)-Caryophyll-5-en-12-al	0.00	0.00	0.00	0.00
1647	1643	Cubenol	0.05	0.07	0.01	0.29
**1650**	**1651**	**α-Muurolol**	**1.41**	**2.12**	**0.56**	**5.65**
1658	1655	α-Eudesmol	0.00	0.00	0.01	0.32
1658	1655	α-Cadinol	0.04	0.00	0.00	0.00
1658	1656	β-Eudesmol	0.00	0.06	0.00	0.00
1660	1660	Selin-11-en-4α-ol	0.35	0.00	0.00	0.00
1671	1666	14-Hydroxy-9-*epi*-(*E*)-caryophyllene	0.00	0.00	0.00	0.28
1681	1676	Bornyl 8-hydroxyisobutanoate	0.00	0.00	0.00	0.14
2052	2050	Bornyl hydrocinnamate	0.00	0.00	0.00	0.00
2151	2152	3-Phenylpropyl 3-phenylpropanoate	0.01	0.00	0.00	0.00
**2224**	**2223**	**Bornyl cinnamate**	**0.59**	**0.42**	**0.27**	**0.16**
2318	2321	3-Phenylpropyl cinnamate	0.03	0.12	0.20	1.00
2434	2432	Phenylallyl cinnamate	0.00	0.00	0.00	0.19
		Monoterpene hydrocarbons	44.63	41.96	82.08	13.32
		Oxygenated monoterpenoids	2.95	7.58	7.29	2.13
		Sesquiterpene hydrocarbons	45.97	42.50	8.68	65.64
		Oxygenated sesquiterpenoids	4.96	6.12	1.22	15.59
		Benzenoid aromatics	0.90	1.24	0.67	1.37
		Others	0.00	0.00	0.00	0.00
		Total identified	99.41	99.40	99.94	98.05

RI_{calc}: Retention indices determined with respect to a homologous series of *n*-alkanes on a ZB-5ms column. RI_{db}: Retention indices from the databases. Compounds in **boldface** were used in the multivariate (HCA and PCA) analyses.

Table S2. Enantiomeric distribution, (+)-enantiomer% : (–)-enantiomer%, of monoterpenoids in *Liquidambar formosana* oleoresin essential oils.

	Compounds				
Sample	α-Thujene	α-Pinene	Camphene	β-Pinene	Sabinene
Re190401A	(+)100 : (–)0	(+)9.4 : (–)90.6	(+)0 : (–)100	(+)10.6 : (–)89.4	(+)100 : (–)0
Re190401C	(+)100 : (–)0	(+)14.0 : (–)86.0	(+)0 : (–)100	(+)18.0 : (–)82.0	(+)100 : (–)0
LD190910C	(+)100 : (–)0	(+)22.9 : (–)77.1	(+)0 : (–)100	(+)7.0 : (–)93.0	(+)100 : (–)0
LD190910D	(+)100 : (–)0	(+)34.9 : (–)65.1	(+)0 : (–)100	(+)14.8 : (–)85.2	(+)100 : (–)0
LD190910E	(+)100 : (–)0	(+)15.2 : (–)84.8	(+)0 : (–)100	(+)4.8 : (–)95.2	(+)100 : (–)0
LD190910F	(+)100 : (–)0	(+)21.0 : (–)79.0	(+)0 : (–)100	(+)16.1 : (–)83.9	(+)100 : (–)0
LD190910H	(+)100 : (–)0	(+)21.5 : (–)78.5	(+)0 : (–)100	(+)31.4 : (–)68.6	(+)100 : (–)0
LD190910I	(+)100 : (–)0	(+)11.2 : (–)88.8	(+)0 : (–)100	(+)14.6 : (–)85.4	(+)87.8 : (–)12.2
LD190910J	(+)100 : (–)0	(+)18.2 : (–)81.8	(+)0 : (–)100	(+)5.3 : (–)94.7	(+)100 : (–)0
LD190910K	(+)100 : (–)0	(+)8.1 : (–)91.9	(+)0 : (–)100	(+)8.6 : (–)91.4	(+)100 : (–)0
LD190910L	(+)100 : (–)0	(+)5.4 : (–)94.6	(+)0 : (–)100	(+)3.7 : (–)96.3	(+)84.9 : (–)15.1
LD190910M	(+)100 : (–)0	(+)34.9 : (–)65.1	(+)0 : (–)100	(+)14.8 : (–)85.2	(+)100 : (–)0
LD190910N	(+)100 : (–)0	(+)4.5 : (–)95.5	(+)0 : (–)100	(+)43.3 : (–)56.7	(+)100 : (–)0
LD190910O	(+)100 : (–)0	(+)12.2 : (–)87.8	(+)0 : (–)100	(+)4.9 : (–)95.1	(+)85.7 : (–)14.3
LD190910P	(+)100 : (–)0	(+)12.2 : (–)87.8	(+)0 : (–)100	(+)4.9 : (–)95.1	(+)86.8 : (–)13.2
LD190910Q	(+)100 : (–)0	(+)4.4 : (–)95.6	(+)0 : (–)100	(+)2.4 : (–)97.6	(+)100 : (–)0
LD190910R	(+)100 : (–)0	(+)7.2 : (–)92.8	(+)0 : (–)100	(+)7.0 : (–)93.0	(+)100 : (–)0
LD190910S	(+)100 : (–)0	(+)5.0 : (–)95.0	(+)0 : (–)100	(+)3.5 : (–)96.5	(+)100 : (–)0
LD190910T	(+)100 : (–)0	(+)5.4 : (–)94.6	(+)0 : (–)100	(+)5.4 : (–)94.6	(+)100 : (–)0
LD190910U	(+)100 : (–)0	(+)6.1 : (–)93.9	(+)0 : (–)100	(+)3.4 : (–)96.6	(+)68.2 : (–)31.8
LD190910V	(+)100 : (–)0	(+)4.4 : (–)95.6	(+)0 : (–)100	(+)3.3 : (–)96.7	(+)100 : (–)0
LD190910W	(+)100 : (–)0	(+)21.5 : (–)78.5	(+)0 : (–)100	(+)31.3 : (–)68.7	(+)100 : (–)0
LD190910X	n.d.	(+)29.6 : (–)70.4	(+)0 : (–)100	(+)24.6 : (–)75.4	n.d.

[a] n.d.: Not determined; concentration too low.

Table S2. Continued.

	Compounds					
Sample	Limonene	Camphor	Terpinen-4-ol	Borneol	α-Terpineol	Bornyl acetate
Re190401A	(+)0 : (–)100	(+)100 : (–)0	(+)36.0 : (–)64.0	(+)0 : (–)100	(+)6.7 : (–)93.3	n.d.[a]
Re190401C	(+)0 : (–)100	(+)100 : (–)0	(+)48.1 : (–)51.9	(+)0 : (–)100	(+)13.4 : (–)86.6	n.d.
LD190910C	(+)0 : (–)100	(+)100 : (–)0	(+)20.2 : (–)79.8	(+)0 : (–)100	(+)8.0 : (–)92.0	(+)0 : (–)100
LD190910D	(+)0 : (–)100	(+)100 : (–)0	(+)38.2 : (–)61.8	n.d.	(+)16.2 : (–)83.8	(+)0 : (–)100
LD190910E	(+)0 : (–)100	(+)100 : (–)0	(+)18.6 : (–)81.4	(+)0 : (–)100	(+)6.9 : (–)93.1	(+)0 : (–)100
LD190910F	(+)0 : (–)100	(+)100 : (–)0	n.d.	(+)0 : (–)100	n.d.	(+)0 : (–)100
LD190910H	(+)0 : (–)100	(+)100 : (–)0	n.d.	(+)0 : (–)100	n.d.	(+)0 : (–)100
LD190910I	(+)0 : (–)100	(+)100 : (–)0	n.d.	(+)0 : (–)100	n.d.	(+)0 : (–)100
LD190910J	(+)0 : (–)100	(+)100 : (–)0	(+)26.0 : (–)74.0	(+)0 : (–)100	n.d.	(+)0 : (–)100
LD190910K	(+)0 : (–)100	(+)100 : (–)0	n.d.	(+)0 : (–)100	n.d.	(+)0 : (–)100
LD190910L	(+)0 : (–)100	(+)100 : (–)0	n.d.	n.d.	n.d.	n.d.
LD190910M	(+)0 : (–)100	(+)100 : (–)0	(+)38.2 : (–)61.8	n.d.	(+)16.2 : (–)83.8	(+)0 : (–)100
LD190910N	(+)0 : (–)100	(+)100 : (–)0	n.d.	n.d.	n.d.	(+)0 : (–)100
LD190910O	(+)0 : (–)100	(+)100 : (–)0	n.d.	(+)0 : (–)100	n.d.	(+)0 : (–)100
LD190910P	(+)0 : (–)100	(+)100 : (–)0	n.d.	(+)0 : (–)100	n.d.	(+)0 : (–)100
LD190910Q	(+)0 : (–)100	(+)100 : (–)0	n.d.	(+)0 : (–)100	n.d.	(+)0 : (–)100
LD190910R	(+)0 : (–)100	n.d.	n.d.	n.d.	n.d.	(+)0 : (–)100
LD190910S	(+)0 : (–)100	(+)100 : (–)0	n.d.	n.d.	(+)2.9 : (–)97.1	(+)0 : (–)100
LD190910T	(+)0 : (–)100	(+)100 : (–)0	n.d.	n.d.	(+)20.7 : (–)79.3	(+)0 : (–)100
LD190910U	(+)0 : (–)100	(+)100 : (–)0	n.d.	n.d.	(+)0 : (–)100	(+)0 : (–)100
LD190910V	(+)0 : (–)100	(+)100 : (–)0	n.d.	n.d.	(+)0 : (–)100	(+)0 : (–)100
LD190910W	(+)0 : (–)100	n.d.	n.d.	n.d.	(+)10.5 : (–)89.5	(+)0 : (–)100
LD190910X	(+)0 : (–)100	n.d.	n.d.	n.d.	n.d.	(+)0 : (–)100

[a] n.d.: Not determined; concentration too low.
